# Advancements in MXene-Polymer Nanocomposites in Energy Storage and Biomedical Applications

**DOI:** 10.3390/polym14163433

**Published:** 2022-08-22

**Authors:** D. Parajuli, N. Murali, Devendra K. C., Bhishma Karki, K. Samatha, Allison A Kim, Mira Park, Bishweshwar Pant

**Affiliations:** 1 Research Center for Applied Science and Technology, Tribhuvan University, Kathmandu 44618, Nepal; 2 Department of Physics, Tri-Chandra Multiple Campus, Ghantaghar, Kathmandu 44605, Nepal; 3 Department of Engineering Physics, AUCE, Andhra University, Visakhapatnam 530003, India; 4 Myrveien 13, Lebesby Kommune, 9740 Lebesby, Norway; 5 Department of Physics, College of Science and Technology, Andhra University, Visakhapatnam 530003, India; 6 Department of Healthcare Management, Woosong University, Daejeon 34606, Korea; 7 Carbon Composite Energy Nanomaterials Research Center, Woosuk University, Wanju, Chonbuk 55338, Korea; 8 Smart Convergence Life Care Research Institute, Woosuk University, Wanju, Chonbuk 55338, Korea

**Keywords:** MXene/polymer, nanocomposites, PCE, drug delivery, microsupercapacitors

## Abstract

MXenes are 2D ceramic materials, especially carbides, nitrides, and carbonitrides derived from their parent ‘MAX’ phases by the etching out of ‘A’ and are famous due to their conducting, hydrophilic, biocompatible, and tunable properties. However, they are hardly stable in the outer environment, have low biodegradability, and have difficulty in drug release, etc., which are overcome by MXene/Polymer nanocomposites. The MXenes terminations on MXene transferred to the polymer after composite formation makes it more functional. With this, there is an increment in photothermal conversion efficiency for cancer therapy, higher antibacterial activity, biosensors, selectivity, bone regeneration, etc. The hydrophilic surfaces become conducting in the metallic range after the composite formation. MXenes can effectively be mixed with other materials like ceramics, metals, and polymers in the form of nanocomposites to get improved properties suitable for advanced applications. In this paper, we review different properties like electrical and mechanical, including capacitances, dielectric losses, etc., of nanocomposites more than those like Ti_3_C_2_T_x_/polymer, Ti_3_C_2_/UHMWPE, MXene/PVA-KOH, Ti_3_C_2_T_x_/PVA, etc. along with their applications mainly in energy storing and biomedical fields. Further, we have tried to enlist the MXene-based nanocomposites and compare them with conducting polymers and other nanocomposites. The performance under the NIR absorption seems more effective. The MXene-based nanocomposites are more significant in most cases than other nanocomposites for the antimicrobial agent, anticancer activity, drug delivery, bio-imaging, biosensors, micro-supercapacitors, etc. The limitations of the nanocomposites, along with possible solutions, are mentioned.

## 1. Introduction

MXenes, pronounced as ‘maxenes’ are the nanolayers of transitional metal carbides, nitrides, or carbonitrides. It was invented by Yury Gogotsi and his group at Drexel University, the USA, in 2011. Most materials discovered after the 2D layer graphene remained for academic purposes rather than practical applications, unlike MXene [1]. The optical, electrochemical, electronic, and mechanical properties of MXenes are optimized by selecting parent compounds, surface chemistry, and intercalating organic compounds to produce a wide array of novel materials with a wide range of applications [2]. They have a layered hexagonal structure (P63/mmc) with strong Van der Waals bonding between layers. They are prepared by a strong solution containing fluoride ions like hydrofluoric acid [2], ammonium difluoride [3], and a mixture of hydrochloric acid and lithium fluoride [4] on the MAX phase with the chemical configuration of M_n+1_AX_n_, in which M is a transition metal, A is A group element, X is to C or N or CN with n equals 1, 2, or 3. MXT_x_ is obtained after etching. Here, T_x_ is a functional group (e.g., O, F, OH) that can be removed by sonication [2]. They become hydrophilic in the absence of hydroxyl or oxygen on their free surface [2,5,6]. MXene is also synthesized by using molten salts (like zinc chloride). However, MXene with chlorine termination is stable up to 750 °C. The development of MXene is reaching up to its large members, including their ordered double transitional metal layered carbides structures with topological insulating properties [7]. They are realized experimentally and theoretically [5,6]. Though the MXenes are highly efficient in many fields, it has some limitations in the physiological surroundings with respect to their stability, controlled nanomechanisms like drug delivery, biodegradability, etc. 

On the other hand, polymers are such types of materials found abundantly in natural and synthetic forms in living (organic: solid parts of all the plants like cellulose, lignin, resin, etc. and animal constituents like proteins, nucleic acids, etc.) and nonliving (Inorganic: diamond, graphite, concrete, glass, paper, plastics, etc.) things. When repeated, a chemical compound or a monomer gives rise to a polymer. Polymers comprising different types of monomers are called copolymers, and those of the same types are called homopolymers [8]. The polymers, in their pure form, have many limitations for their use in advanced devices. To get unmatchable properties, we can mix different nanoparticles with them to form hybrid polymers or nanocomposites. They can be used in different types of sensors, biomedics, energy storage, etc. [5]. Sensors play an important role in biomedical and energy storage mechanisms under our study. The fundamental parameters that play an effective role in sensors are their electrical conductivity, flexibility, sensitivity, and repeatability. They are discussed briefly with their working formulae, equally important to MXene Polymer nanocomposites, in Section 5. 

The highest modulus, solubility in an organic solvent, and nanoparticle compatibility in 2D materials made MXene the best nanocomposite agent. The main advantages of the MXene polymer against other polymer composites related to our concerned fields are shown in the last section. 

In this paper, we will try to focus on the nanocomposites made by the polymer and MXene to overcome their limitations in their individual state and enhance their conversion efficiency, bio and physicosensitivity, and antibacterial activity, and so on. Initially, before 2000, the study was focused on composite. After the prevalence of fillers of size less than 100 nm, these types of composites were found to have higher performances in most of their properties due to characteristic features of nanosizes, thereby widening the field of the nanocomposites [6]. Previously, most reviews were done on titanium carbide-based MXenes [9]. Here, we have added more MXenes with several other polymers than in the recent reviews [10,11], which are described in Section 3. The formation of hybrid materials or the nanocomposite will supplement their properties with each other for a better resultant product. The formation of nanocomposites enhances their mechanical properties, structural and thermal stability, electrical conductivity, noise damping, corrosion resistance, low permeability of fluids, lower density, low filler content, and ease of manufacturing. On the other side, the nonuniform distribution, higher viscosity, and formation of agglomeration are their limitations [12]. We are trying to discuss biomedical and energy briefly. The brief background on the targeted fields is as below.

### 1.1. Biomedical 

The biomedical application of Ti_3_C_2_-based MXene is crucial in the biomaterial market in treating, diagnosing, or augmenting tools [13] derived from polymer, ceramic, metal, or composite materials [14]. Further, nano-medicine-based applications are more pronounced these days. The ceramic-based MXenes are the most desired material for biocompatible accessories [15]. They are conducting in metallic range, tunable, absorbents, and non-toxic to living beings. However, they are less stable in the outer environment, and it is hard to control their drug delivery flow, affecting the healthy cell or tissues, etc. The formation of a composite overcame these limitations with different biodegradable polymers like polyvinylpyrrolidone (PVP) [16,17] polyethylene glycol (PEG) [18], cellulose [19], polyvinyl alcohol (PVA) [16], soybean phospholipid (SP) [17,20,21,22,23] or inorganic nanoparticles such as polyoxometalates (POMs) [24], mesoporous silica nanoparticles (MSNs) [25], etc. which reduce the cost and pain that is left behind stitching [26]. The composites are also used as photothermal agents [27]. The terminations on MXenes affect their electronic behavior, which is mainly semiconductors or metallic [28,29], thereby imparting antibacterial property and wastewater treatment [30,31] cancer diagnostic and treatment [17,20,21,22,27] photothermal therapy (PTT) via hyperthermia, synergistic PTT/chemotherapy, etc. [17,20,21,22,27]. Antibacterial activity, drug delivery, photothermal therapy, tissue culture, bio-sensing, bio-imaging, etc., are some advanced properties of MXene. Besides polymer nanocomposites, WO_3_/MXene was another composite synthesized by a hydrothermal approach and characterized their structural, morphological, spectral, elemental, and superficial properties. The photocatalytic degradation of MXene, WO_3_/MXene, and WO_3_ were 54%, 89%, and 99% respectively. Thus, WO_3_ addition increases the PD capacity of MXene. Likewise, the antibacterial agent against positive and negative strains was found to be good and concentration-dependent respectively. The inhibition zone of the nanocomposites was increased from 7 to 9 mm with concentration. This is another best example of the improvement of MXenes’ property with the formation of nanocomposites [32]. The MXene composites with abundantly and daily used ferrites material [33,34,35,36,37,38,39] are under study.

### 1.2. Energy Storage

MXenes are very good in energy storage. The addition of suitable components to form nanocomposites can enhance their storage capacity. The storing capacity depends on conductivity, cyclability, specific capacitance, capacitive retention, etc. Some of the MXenes exotic for lithium-ion batteries are V_2_CT_x_ [40], Nb_2_CT_x_ [40], Ti_2_CT_x_ [41], and Ti_3_C_2_T_x_ [28]. Ti_3_C_2_T_x_ MXene has high electrical conductivity, 2.4 × 10^5^ Sm^−1^, as high as that of multi-layered graphene. These types of MXenes have a good response with the RF fields and for charging and discharging phenomena. Conductive polymers under organic materials differ from inorganic solids with tunable electronic, optical, stable, and conducting properties [29,42,43]. They are exotic in various applications like in smart windows [44], thermoelectrics [45], biosensors [46], corrosion protection [47], light-emitting diodes [48] and energy storage devices [49,50,51]. The in-situ electrochemical process is one of the effective methods for synthesizing such materials [52,53,54,55,56]. The composites of organic-inorganic nanocomposite cannot be synthesized easily by a one-step electroplating (EP) method. In one step EP, the highly conductive solution is mixed with the polymer, to form high-performance solid-state micro-supercapacitors (MSCs). This limitation can be overcome by using the MAX phase with in-plane vacancy ordering termed *i-MAX* [57,58]. Mo_1.33_C is such a highly conducting MXene capable of supercapacitor applications [50,51] which, when mixed with conducting polymer [51], are widely used as plates for the capacitor for efficient energy storing. The electroplating is replaced by a novel in situ electrochemical polymerization process to prepare 2D MXene-doped conductive polymer films. Mahmood et al. in 2021 found Vanadium Pentoxide with an efficient electrode material, which was not practical due to its lower electrical conductivity. This was overcome with the formation of nanohybrids with MXene, which resulted in a specific capacity of 768 F/g (at 1 A/g), a specific capacity of 93.3% after 6000 Galvanostatic Charge Discharge (GDC) test, increased current density from 1 to 5 A/g, etc. due to the properties of MXenes [59].

### 1.3. Sensors

It became necessary to discuss the sensors as they play an important role in both biomedical and energy storage mechanisms. The repeatability, reproducibility, sensitivity, and selectivity for a longer time are the major components of any sensor for their efficient performance [60]. MXene is one of the most sensitive gas sensors to date [61]. This is due to their attached terminations which are affected even with a very small amount of gases. They can identify ammonia and acetone-like chemicals, indicators of ulcers and diabetes. The layered chemical structure and porous membrane of MXenes are other unique structures for their performance. Various MXene sensors include electrochemical, gas, stress-strain, photoluminescence, etc. The flexibility can be added to such sensors to produce flexible humidity, VOC, mechanical, and parametric sensors [62]. The MXene polymer-based nanocomposites used for the sensors are much more efficient than the conductive polymer nanocomposites used for the same purposes and are discussed in Section 5 [60].

This review work will reveal a brief idea of the synthesis and structure of MXene, different types of polymer MXene nanocomposites, and their applications in the biomedical and energy storage field. The review was necessary as two major fields, medicine and energy, directly affect daily human life. Further, many advanced technologies and materials are being developed in those fields. The most exotic material, MXene, is more biocompatible and superior in energy-storing but was limited by its flexible strength, which was enhanced with the formation of nanocomposites with polymer. 

## 2. Synthesis and Structure of MXene 

**Figure 1:**
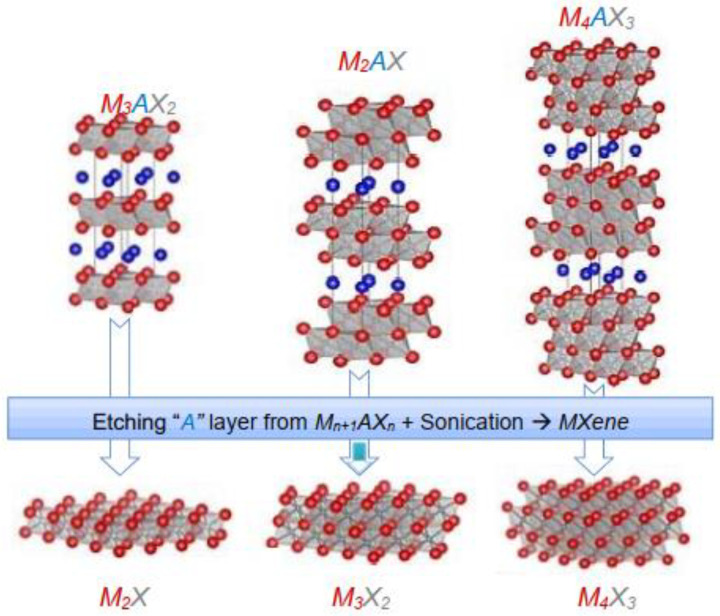
Schematic figure with crystal structure showing the conversion of MAX (**top**) to MXene (**bottom**). Reprinted with permission from Reference [2].

**Figure 2:**
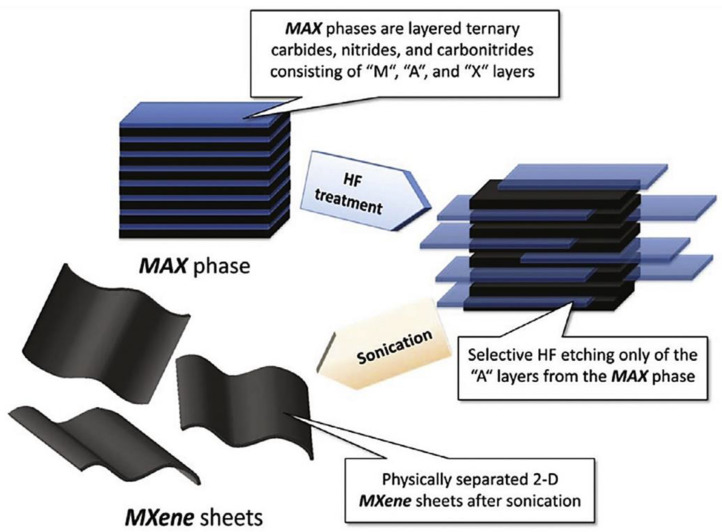
Wet-chemical etching in hydrofluoric acid. (Reproduced with permission [1]. Copyright 2012, American Chemical Society.).

MXenes have M_n+1_X_n_ composition obtained after etching out A element from their parent MAX phase with composition M_n+1_AX_n_, where ‘M’ denotes an early transition-metal, ‘A’ group 13 or 14 element, ‘X’ carbon or nitrogen, and n = 1, 2, 3 [2]. The etching methods are of three types: (a) molten salt treatment method, (b) fluorination treatment, and (c) wet chemical etching. The schematic diagram of the crystal structure of the MAX to MXene phase and the preparation of MXene by wet chemical etching is shown in Figure 1 and Figure 2, respectively. The figures show the mixing of MAX phase powder with aqueous HF and stirring sufficiently for its proper etching. The MXene sheets were obtained after the sonication of the etched MAX precursor [1]. 

The MXene obtained were with functional groups T_x_-hydroxyl (-OH), oxygen (-O), and/or fluorine (-F), which were removed with sonication by ultrasonicator [20]. The terminations have a crucial role in providing hydrophilicity and surface modification by PVP (polyvinyl pyrrolidone), SP (soybean phospholipid), PVA (polyvinyl alcohol), PEG (polyethylene glycol), PLGA (poly lactide-co-glycolide) to enhance the physicochemical stability and biocompatibility of the MXenes [24,28] especially important for polymer MXene nanocomposites. There are millions of possible arrangements of transitional metals (like titanium, molybdenum, etc.), carbon, and nitrogen to produce stable MXenes. They have a unique porous structured, layered membrane that can trace even very low concentrations of molecules. Unlike graphene, MXenes have a wider band gap which can be exploited for several advanced electronic devices. For example, the band gap of some MXenes Oxides like Hf_2_CO_2_, Zr_2_CO_2_, and Ti_2_CO_2_ are found to be 2.45, 2.13, and 1.15 eV, respectively. Hf_2_-_2x_Ti_2x_CO_2_ (0 ≤ x ≤ 1) can have band gap enhanced from 2.45 to 1.15 eV [63]. The list of the band gap of the other MXenes can be found in the previous literature [64]. Another important feature of MXene structure is that their quality and properties can be achieved by controlling, choosing, or preparing appropriate MAX phases. 

**Figure 3:**
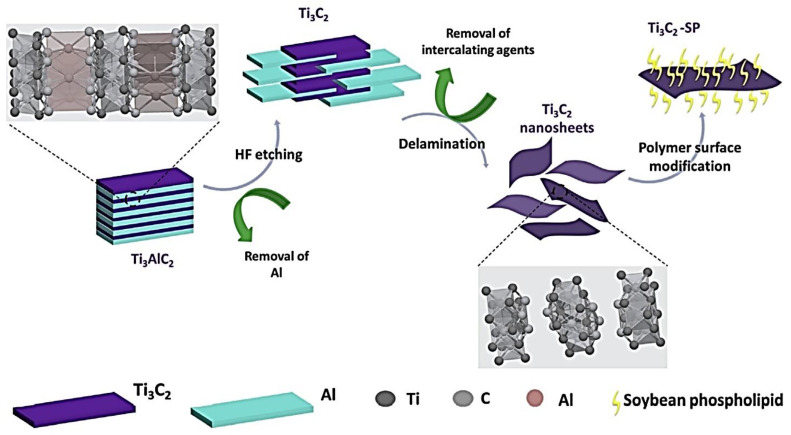
Synthesis cycle of Ti_3_C_2_-SP nanocomposite [11]. Reprinted with permission from Ceramics International. © 2019 Elsevier Ltd. and Techna Group S.r.l.

Alternatively, tetrapropylammonium hydroxide (TPAOH) intercalation [27,65] can be done for delamination and without harmful fluorine for etching [66]. The MXene-SP composite synthesis cycle is shown in Figure 3 schematically. The bottom-up approach can also be utilized to synthesize MXene by EVD-like techniques [65].

**Figure 4:**
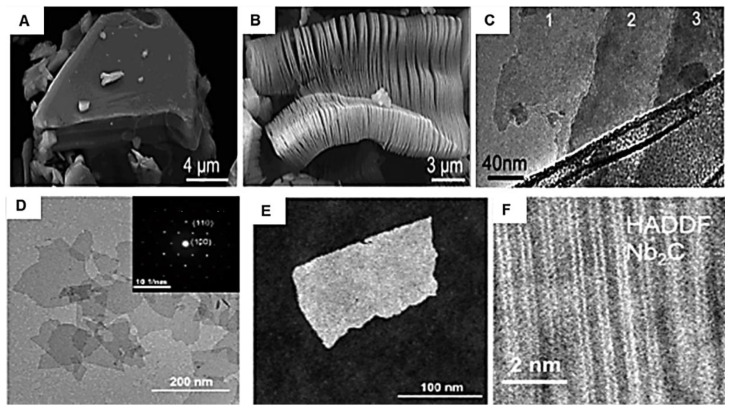
(**A**) SEM micrographs of Ti_3_AlC_2_ before and (**B**) after etching. (**C**) TEM images of Ti_3_C_2_ after etching [1] © American Chemical Society (2012). (**D**,**E**) TEM images (bright and dark field Nb_2_C). (**F**) High-resolution HAADF-STEM image. Adapted from Reference [12] © American Chemical Society (2019).

The interlaced MA bond in MAX is weaker than the MX bond. Thus, A can be easily etched out with X in an octahedral site [67] with terminations attached with hydrogen bond and Van der Waals forces [2]. The high-resolution transmission electron microscope (HRTEM), selected area electron diffraction (SAED), high angular annular dark field (HAADF), and scanning transmission electron microscopy (STEM) images in Figure 4 shows layered intercalated nanosheets of the hexagon with space group of P63/mmc ‘n’ dependent atomic arrangement with lateral size in the range 0.5 to 200 nm and thickness of few nanometers.

## 3. MXene Nanocomposites

MXenes can be effectively mixed with other materials like ceramics, metals, and polymers in the form of nanocomposites to improve (electronic, magnetic, mechanical, thermal, optical, structural, etc.) properties needed for the advanced application. The predominant MXene-based nanocomposites are MXene/polymer composite, MXene/metals/ceramics composite, MXene/carbon composite, MXene-based hydrogels, etc. They can be fabricated by hydrothermal/solvothermal, solution processing, drop-casting, adsorption, hot pressing, in situ polymerization blending methods, etc. The oxides of metals, graphene derivatives, polymers, etc., are abundantly found in composition with MXene. A brief description of some MXene nanocomposites is as follows:

### 3.1. MXene/Metals/Ceramics Composites

The catalytic nature, electrical conductivity, and mechanical strength of metals in combination with hydrophilic surfaces and mechanical strength of MXene give rise to a new form of composite which manages the agglomeration and poor wettability of metals [68]. Thus, the symbiotic effect in the composite generates the stacking of layers on oxides and sulfides [69]. 

### 3.2. MXene/Carbon Nanocomposites

Carbon-based materials like nanotubes, fibers, graphene, etc., have rich optical, electronic, structural, and mechanical properties incorporated with MXene adding flexibility, prosperous electrochemical properties, and conductivity to the resultant nanocomposites [70]. The graphene layer stacking is done without a reduction in electrical and surface area properties [71]. The electron pathway is created as a 3D network structure when carbon nanofiber is in the form of a composite. The numerous electron holes in the CNFs contribute to the electrochemical properties and decrease the material’s resistance. The heterojunction between graphene/Mo_2_C and Cu/Mo was fabricated by Xu et al. using carbon from methane gas through the CVD process [72]. The introduction of Ti_3_C_2_T_x_/CNTs in between the MXene layers by the CVD method gives rise to the composite with a superb EM wave absorber with a 99.99% frequency absorber (4.46 GHz). The variation in thickness can improve the frequency range absorption up to 14.54 GHz. 

### 3.3. MXene Hydrogels 

**Figure 5:**
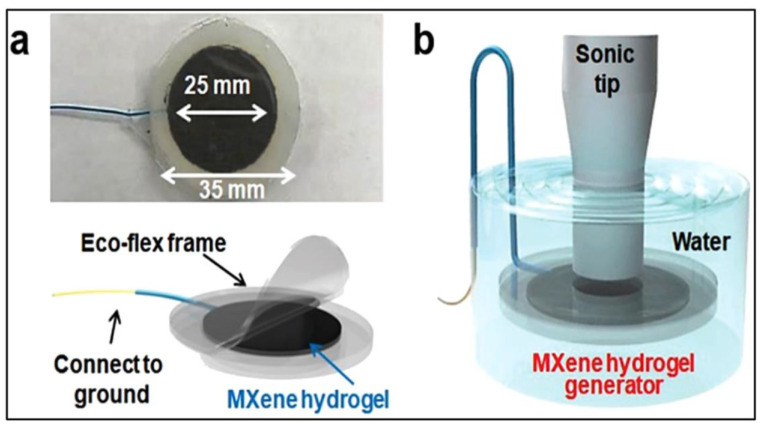
A simple MXene PVA-hydrogel Generator Setup Sketch (**a**) a thin hydrogel fixed between two ecoflex covers (**b**) the sonic tip generating output voltage. Reprinted with permission from [76], copyright 2020, American Chemical Society.

The 99% water and the remaining 1% gelating substance like polymers, solvated or inorganic particles give rise to the hydrogels. They have physicochemical and rheological characters like solids on a macroscale. Most of the precursors are in the form of hydrogels [73]. When mixed with hydrogels, nanoparticles respond to optical, acoustic, electrical, conductivity, hydrophilicity, and tunable properties [74]. As a result, they are used in energy storage, biomedical applications, catalysis, electromagnetic interference shielding, sensing, energy harvesting, etc. Among different applications, energy harvesting for biomedical applications is much more interesting, promising, and important. The devices like pacemakers and neurostimulators need long terms operation (dozens of years) charging with a sustainable energy source. Such energy can be compensated by ultrasound waves received through human tissues. This mechanism can be operated with the use of a simple device. The schematic diagram of a simple PVA-hydrogel generator for energy harvesting is shown in Figure 5. Figure 5a shows a thin hydrogel is fixed between two ecoflex covers. Figure 5b shows the sonic tip in contact with the device, generating an output voltage of around 2.8 V [73]. The efficiency of hydrogels can be enhanced with the use of nanofillers in hydrogels along with the MXene. The resultant provides self-healing, stretchability (more than 3400%), and has mechanical sensitivity ten times more than the pure hydrogel [65]. However, the metastable states in MXenes have pros and cons in the hydrogel performance [75]. 

## 4. MXene-Polymer Composites 

There are various MXene-polymer nanocomposites. A brief idea of some nanocomposites are described as follows:

### 4.1. Polyvinyl Butyral Composite of MXene

The reaction between aldehyde and alcohol with Ti_3_C_2_T_x_ MXene gives rise to acetal natured transparent polyvinyl butyral (PVB). They are used as a resin in the glass industry and then in vehicles, tall buildings, and decoration [77]. Yang et al., 2017 developed a PVB/Ba_3_Co_2_Fe_24_O_41_/Ti_3_C_2_ composite with the capacity to absorb in the wider frequency range [78] that absorbs the EM wave with R_Lmax_ value −46.3 dB at a frequency of 5.8 GHz and R_Lmin_ value −10 dB at 1.6 GHz. Thus, this composite is used as EMI shielding in RADAR, etc. 

### 4.2. UHMWPE Composite of MXene

The mixture of Ti_3_C_2_T_x_ and Ultra-High Molecular Weight Polyethylene (UHMWPE) gives rise to a composite as thermoplastic polyethylene having a long chain character [79], which can be modified to strong thermoplastic with pressure and heat. Their anti-corrosive, low abrasive, and moisture-resistant character made UHMWPE more useful [78]. The increased Ti_3_C_2_ concentrations enhance their mechanical properties. Simultaneously, the crystallinity increases with decreasing the coefficient of friction. Further, analogous to Graphene, it acts as a lubricator [80]. Thus, the composite has improved properties than the pure MXene. Given their lower moisture absorption ability, they can reject moisture applicable to conveyor belts, marine equipment, food processing, etc. 

The tribological [81,82] and mechanical properties of polymers [9] can be enhanced by the addition of MXenes (Ti_3_C_2_) to form Ti_3_C_2_/polymer composites. Among them, ultra-high molecular weight polyethylene (UHMWPE) is resistive, lubricative, and chemically inert despite limitations in mechanical properties [83,84]. Adding inorganic fillers like ZnO, Al_2_O_3_, carbon nanotubes, and graphene improves their properties like tribological, thermal, mechanical, etc. The use of graphene is also practiced in such composites [85,86,87]. 

### 4.3. PES Composite of MXene 

Polyethersulfone (PES) is an amorphous thermoplastic ability to tolerate high temperatures in water and air. In addition to the higher thermal resistance, it has chemical, mechanical, electrical, and optical properties [88], which are significant in desalination and wastewater treatment [89]. Runlin Han et al., 2017, separated Congo red dye and inorganic salts to form an excellent hydrophilic MXene/PES composite. It is found that the dye is rejected 80.3% for the flux of about 117.69 Lm^−2^h^−1^ and 10.7% for 114.9 Lm^−2^h^−1^. They are used in ultrafiltration membranes for wastewater treatment [90].

### 4.4. CS Composite of MXene 

Chitosan (CS) is an amino polysaccharide. The chitin of insets crustaceans after deacetylation gives rise to chitosan. They have antioxidant and antibacterial properties that have cholesterol and triglyceride trapping effects [91]. Liya Zhou et al. formulated an acetylcholinesterase/chitosan/MXene-based biosensor to identify the presence of organophosphate in water and food. Similarly, AChE/CS-Ti_3_C_2_T_x_/GCE biosensors were developed coating AChE over GCE after dipping Ti_3_C_2_T_x_ nanosheets in 0.20% CS solution. The presence of CS-Ti_3_C_2_T_x_ shows its high electrocatalytic activity, enhancing the degree of electron transfer between the electrolyte cells. This process was checked after adding AChE due to the non-conductive enzyme property. In their study, the recovery of malathion with a 94–105% range indicates its biosensing property [83]. Hence, these MXene-polymer biosensors are lightweight and flexible with updated properties with a reliable detection rate. Similarly, this composite can detect the glucose level in diabetic patients, pollution monitoring, food processing, etc. 

### 4.5. CNF Composite of MXene 

Cellulose nanofiber (CNF) are thin fibers of wood materials that provide hardness in plants. So, CNFs are used as building bio-materials [84,92]. Wen-Tao Cao et al. used a vacuum filtration-supported self-assembly process to fabricate an MXene/CNF paper. Its ultra-thin and anti-fracture strain with nacre-inspired structure flexibility with higher tensile strain were suitable for EMIS. The shielding effectiveness was ~25.8 dB at 12.4 GHz with 80% of d-Ti_3_C_2_T_x_ and electrical conductivity of ~739.4 Sm^−1^ [93]. 

### 4.6. PS Composite of MXene 

Polysulfides (PS) are elastic under the synthetic rubber family. They are used for sealing and sticking in the automotive and construction industry [94]. The appropriate use of lithium polysulfides (Li_2_S_n_) in electrolytes gives rise to Li-S batteries as one of the solutions for energy storage devices studied by Yuming Zhao’s group using density functional theory and found the improved electrochemical properties [95]. Xiao Liang et al., 2015 focused on the higher concentration of sulfur (S) and improved the supercapacitor materials. They have a capacity reduction of 0.05%/cycle, the SC of 1200 mAhg^−1^ over 5 h. C/DC current rate, and a CRR of 80% over 400 cycles at 2 h. C/DC current rate [96]. 

### 4.7. PVDF Composite of MXene 

Poly (vinylidene fluoride) (PVDF) is a fluorine plastic with higher dielectric values fused at 373 K used for electrical insulation and decorative coating [97]. Kashif Rasool et al. mixed PVDF’s ultrafiltration quality with MXene and investigated the antibacterial rate. It was 73% and 67% of pure PVDF membrane when tested with B. subtilis and E. coli, respectively. The TiO_2_/C is formed on the surface of the aged membrane with 99% growth inhibition. Wastewater can be treated with their support [36]. Renyuan Li et al. studied a stacked MXene layer deposited on hydrophilic PVDF and found the resulting material photothermally compatible for advanced application [98]. Shaobo Tu et al., 2018, studied the MXene dispersed in P(VDF-TrFE-CFE) and found the enhanced dielectric permittance 10^5^ for 10%wt. MXene [99]. This is very important in capacitors and rechargeable batteries. 

### 4.8. PPy Composite of MXene 

Polypyrrole (PPy) and heterocyclic polymer polypyrrole (PPy) prepared in 1968 have good electric, electronic, and mechanical properties [100,101]. Wenling Wu et al. prepared organ-like Ti_3_C_2_/PPy nanocomposites for supercapacitor electrode materials. They have specific resistance of 184.36 Fg^−1^ at 2 mVs^−1^ under 83.33% CR after 4000 cycles at 1 Ag^−1^ showing this new composite to be an electrode material of supercapacitor with promising electrochemical performance [102]. Minshun Zhu et al. fabricated a composite by intercalating PPy into layered MXene. A 203 mF cm^−2^ capacitance with the retention of 100% even after 20,000 charging/discharging cycles was reported [103], showing their promising electrochemical properties in flexible supercapacitors. 

### 4.9. P(VA)/P(AA) Composite of MXene 

Xinxin Huang et al. intercalated DMSO with MXene at STP, resulting in poly (vinyl alcohol)/poly (acrylic acid)/Fe_3_O_4_/MXene @Ag nanocomposite whose catalytic activity was suitable for wastewater treatment. Ag-based catalytic activity help in the reaction of nitro compounds [104]. Similarly, TiO_2_ nanoparticles, already a photocatalysis, when mixed with Ti_3_C_2_/PVA/PAA, may enhance antibacterial activity by diluting sulfuric and nitric acid, reducing the corrosive effect in the plant equipment.

### 4.10. PDMAEMA Composite of MXene 

Poly (2-(dimethylamino) ethyl methacrylate) (P(2(DMA)EMA) is another biocompatible polymer composite of MXene showing excellent performance in drug delivery like insulin oral delivery. Proteins and nucleic acids are bioactive polymers that can pacify the positive charge when reacting with PDMAEMA [105,106]. In the work of Jing Chen et al. in 2014, the incorporation of V_2_C with PDMAEMA shows nice tuning in transmittance and conductivity by the increment of conductivity and transmittance from 2.8 to 33.7 mS cm^−1^ and 15% to 75% along with the rise of the temperature from 25 °C to 45 °C, respectively. The temperature change may change the hydrophilic to the hydrophobic property of V_2_C@PDMAEMA. The carbonic acid, resulting from CO_2_ and H_2_O, is diluted with the increase in conductivity [107]. CO_2_ and H_2_O concentration measure the toxicity of in-vivo sensors. 

### 4.11. PU Composite of MXene 

Polyurethane (PU) can check the problems due to weathering, solvents, and mechanical damages because of their biocompatibility and hence used in pacemakers and artificial hearts [108,109]. Similarly, tall buildings use thermoplastic polyurethane (TPU), which is cheap and easily available. However, they release harmful gases when on fire. Bin Yu et al. used surface terminations of the MXene mixing with tetrabutyl phosphine chloride (TBPC) and cetyltrimethylammonium bromide (CTAB), respectively, which are safer (later more) in a fire than normal polymer [110]. The MXene was incorporated with polymer by Weiqiang Zhi in 2018 with the emulsion method, after which the mechanical properties of PU were increased sharply [111], which are widely used in pacemakers. They are also used in energy storage.

### 4.12. PANI Composite of MXene 

Polyaniline (PANI) is a highly conductive ancient polymer used in conductive coating [112,113]. Huawei et al. prepared a Ti_3_C_2_T_x_/PANI composite to study what content they absorb in microwaves. The absorption efficiency of 99.999% with Rl of −56.3 dB at 13.80 GHz was achieved with a sample mass ratio of 1:3 [114]. They are portable and tunable sources for wearable electronics [115]. Similarly, extraordinary conductivity, capacitance, and performance were seen in Ti_3_C_2_T_x_/poly (3,4-ethylene dioxythiophene): polystyrene sulfonate (PEDOT: PSS) fibers prepared by Jizhen Zhang et al., which were better than the other MXene hybrid fibers [116]. It is used in electric insulator [117]. Xinxin Cao et al., 2017, prepared MXene/LLDPE nanocomposite and found better thermal stability of composites than pure MXene [118]. 

### 4.13. Poly-3,4-ethylene Dioxythiophene Composite of MXene 

Chi Chen et al. showed the effect of charge transfer in the polymerization of ethylene dioxythiophene (EDOT) intercalated within the MXene layers. Its electrochemical property shows that the first charging/discharging cycle is 575 and 307 mAhg^−1^, with the capacitance of 83% sustained even after 100 such cycles. This composite has better performance than the standard lithium-ion battery [119,120]. 

### 4.14. PE Composite of MXene 

Poly (ethylene terephthalate) (PET) is an artificial fiber popularly known as polyester prepared by combining ethylene glycol and terephthalic acid under the pressure and temperature of 400 kPa and 290 °C. Mixing with cotton makes it cheaper and more useful [121]. Wenyu Shao et al. prepared polyester/MXene nanofiber whose morphology was like modified yarn showing the self-winding of the nanofibers around the polymer. They have fabricated a supercapacitor of yarn coated with MXene nanofiber and found a specific capacitance of 18.39 m F cm^−2^ at 5 mV s^−1^ and a high power density rated at 0.39 mW cm^−2^ while maintaining an energy density of 0.38 µW h cm^−2^. The prosper electrochemical and mechanical properties of yarn make its performance of 98.2% retained over an astounding 6000 cycles [122]. These studies show the avenue toward wearable technologies.

### 4.15. PEI Composite of MXene 

Xiaoli Wu et al. incorporated the hydroxyl (-OH) groups as fillers in the polyethyleneimine (PEI)/Ti_3_C_2_T_x_/polydimethylsiloxane (PDMS) hydrophobic polymer where there are PAN/PEI-Ti_3_C_2_T_x_-X: X = 1, 2, 3, 4 and PDMS (PAN/PDMS-Ti_3_C_2_T_x_-Y: Y = 1, 3, 5, and 10) are found as membranes. It was found that the PDMS and PEI membranes are suitable for non-polar and polar solvent systems [123]. They are used as solvent-resistant nanofiltration in alcohol-based mixtures.

### 4.16. PAM Composite of MXene 

Polyacrylamide (PAM) polymers are soft, gelatinous, and protein resistant, hydrophilicity, and biocompatibility [124,125]. M. Naguib et al. mixed PAM and MXene to get higher flexibility and conductivity. This nanocomposite has flakes oriented randomly and can polymerize the layers of MXene in in situ mode. Their conductivity was increased up to 3.3 × 10^−2^ Sm^−1^ when the membrane was incorporated with 6 wt.% MXene [126]. 

### 4.17. GdW10-Based Polyoxometalates Composite of MXene 

Luyan Zong et al. destroyed tumor cells permanently using PPT on GdW10-based polyoxometalates composite solution where Ti_3_C_2_ serves as a contrast agent for improved CT and MR imaging. They are used in biomedical fields [24]. 

### 4.18. PS Composite of MXene 

Polystyrene (PS) is hard plastic with partial flexibility and is colorless, which is mostly used in packaging, plastic cutlery, CD cases, etc. [127,128]. Touseef Habib et al. mixed functionalized MXene (Ti_3_C_2_T_x_) on PS. Di hexadecyl dimethyl ammonium bromide (DDAB), decyl trimethyl-ammonium bromide (DTAB), and octadecyl trimethyl ammonium bromide (OTAB) were used as cationic modifiers resulting in PS/DDAB/MXene, PS/DTAB/MXene, and PS/OTAB/MXene composites. After the formation, 21.5%, 20.8%, and 26.4% of the heat were released, respectively [129]. This shows how the flame is retarded with the MXene polymer composites.

### 4.19. PDDA-Ti_3_C_2_T_x_ Nanocomposite 

**Figure 6:**
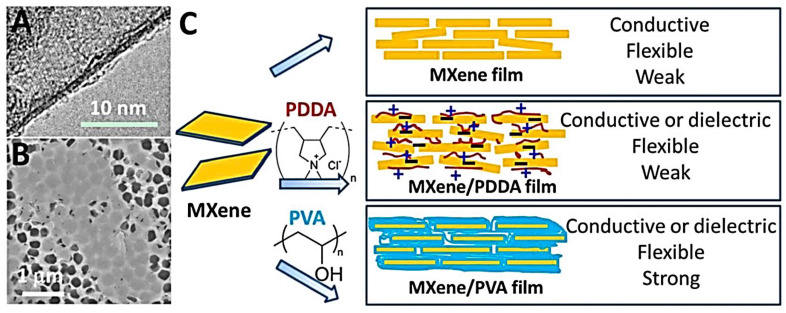
(**A**) TEM and (**B**) SEM images of MXene flakes after delamination and before film formation (**C**) MXene-based functional film schematics [9].

Figure 6A–C shows the TEM micrographs of the delaminated single Ti_3_C_2_T_x_, SEM image before the delamination of the MXene sheets, and the sketch of MXene-based functional films [9]. The vacuum-assisted filtration gives rise to the ordered stacking of Ti_3_C_2_T_x_ flakes along, and the peaks are shifted to a lower angle compared with that of pure Ti_3_C_2_T_x_ films (6.5°) due to the intercalation of PDDA molecules between the Ti_3_C_2_T_x_ flakes [9]. 

Similar to the pure Ti_3_C_2_T_x_, ~2000 S/m of conductivity was obtained by the films with polydiallyldimethylammonium (PDDA). The presence of polymer between the MXene flakes reduces the conductivity. Similarly, Ti_3_C_2_T_x_/PVA films were also freestanding and flexible. Adding PVA increases the composite film’s thickness in the range of 4 to 12 μm. Hence, during the shifting of 0002 peaks toward lower angles in the range of 4.8° to 6.0°, FWHM increases correspondingly, indicating the increase in the interflake distance and decrease in uniformity. The conductivity decreases with the PVA constant and interflake distance of Ti_3_C_2_T_X_. The tensile force is increased four times with the addition of PVA [9]. 

Similarly, the cations intercalation in the MXene layers increases the capacitive performance of the Ti_3_C_2_T_x_-based nanocomposite as a supercapacitor. The Ti_3_C_2_T_x_/PDDA has a lower volumetric capacitance of 296 F/cm^3^ at 2 mV/s than that of pure Ti_3_C_2_T_x_ valued at 3.19 g/cm^3^. In the same way, the Ti_3_C_2_T_x_/PVA/KOH is a gel electrolyte for energy storage [130,131] with capacitances increased by 80% and valued at 2 mV/s higher than the other two. Thus, Ti_3_C_2_T_x_ flakes polymer nanocomposite has exotic mechanical and electrochemical properties. Moreover, they are also used in radiofrequency shielding, water filtration, fillers, wearable, and flexible energy storing devices. 

### 4.20. ePTFE/MXene Composite

Liu et al. recently prepared an expanded polytetrafluoroethylene (ePTFE)/MXene composite. They found enhanced hydrophilicity (water absorbent) and lipophilicity (oil absorbent) properties that were useful in an aquatic environment affected badly by oil leaks. The dual pore structure in the prepared composite made them widely used in absorbing overflow oil and water in our daily life [132].

### 4.21. Alginate/MXene Composite 

Alginic acid or algin ((C_6_H_8_O_6_)_n_), with an acidity of 1.5–3.5, is one form of the natural and edible polysaccharide available in brown algae in the appearance of a white, yellow fibrous powder. Its sodium and calcium-based salts are called alginate. Though there are lots of sorbents based on MXenes, their capabilities are limited. The MXene, when mixed with alginate, gives rise to a composite whose adsorbing capacity is highly enhanced. It helps in adsorbing the Pb^2+^ and Cu^2+^ @ 382.7 and 87.6 mg g^−1^ in 15 min, respectively, from wastewater. In addition, the chelating capacity and ion transport capacity of lead and copper are highly enhanced. The biopolymer alginic acid or algin is useful for several bio applications when suitably combined with electrolytic manganese dioxide (EMD). Its electrochemical performance becomes five times more than its pristine form. Besides MXene components, other biopolymer composites like L-glutamic acid mixed with molybdate [133], alginate biopolymer [134], and CoMoO_4_ mixed cetyltrimethylammonium bromice (CoMoO_4_/CTAB) [135], etc. are found to have robust electrochemical agents. 

### 4.22. Ti_3_C_2_/Cellulose Composite

Xing et al. [19] found Ti_3_C_2_/cellulose composite hydrogel to be a biocompatible, biodegradable, and load bearer for drugs after their photothermal conversion. They can deliver the anticancer drug doxorubicin hydrochloride (DOX) in at least 48 h. They can scavenge and kill cancer/tumor cells by photothermal and chemotherapy at around 50–55 °C. It represents one of the successful experiments in cancer treatment using MXene/polymer nanocomposites.

### 4.23. Lignin/MXene Composite

**Figure 7:**
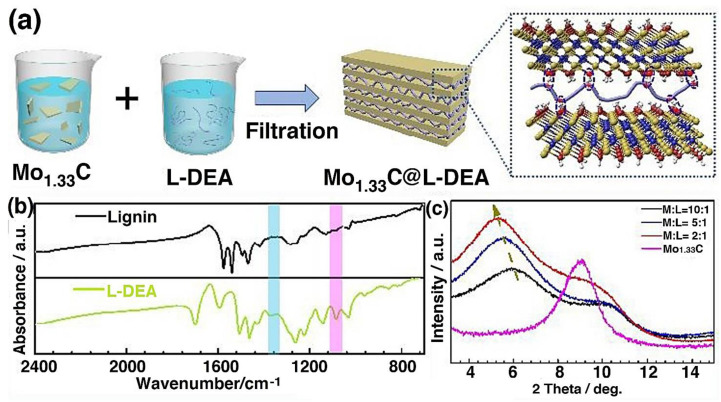
MXene/L-DEA nanocomposites. (**a**) Preparation sketch of the Mo_1.33_C@L-DEA composite electrode. (**b**) FT-IR spectra of the lignin and L-DEA. (**c**) XRD patterns of the hybrids and pure Mo_1.33_C films. Adopted with permission from [136].

The Mo_1.33_C solution and amine cations functionalized Lignin (L-DEA) ink can be mixed to form MXene/(L-DEA) nanocomposites, as shown in Figure 7a. Figure 7b,c shows the FTIR (for Lignin and LDEA) and XRD of the sample (of pure Mo_1.33_C, 10:1, 5:1, 2:1 mass ratio with LDEA), respectively. The peaks are shifting towards a lower angle site indicating the increasing distance between the Mo_1.33_C layers, thereby preventing the restacking of MXene nanosheets. The composite is used as a paper negative electrode and exfoliated graphene with ruthenium oxide (EG@RuOx) as a positive electrode that gives rise to the excellent capacitance of 503.7 F g^−1^, the energy density of 51.9 Wh kg^−1^, and power density of 40,095 kg^−1^ at 35 V [136]. They are used as asymmetric supercapacitors. 

## 5. Applications

Out of many applications of polymer MXene nanocomposites, we have focused on their biomedical and energy storage aspects: 

### 5.1. Biomedical Applications 

The Biomedical applications are mainly based on:

#### 5.1.1. Antibacterial: Antimicrobial Agent & Anticancer Activity

The polymerized MXene is a good medical agent in antimicrobial, photothermal therapy (PTT), drug delivery, diagnostic imaging, biosensors, and bone regeneration. The special properties of MXene are due to its terminations –O, –OH, –F, etc., attached to its surface. These terminations play a vital role in biomedical applications especially in anticancer [23,28], antibacterial [137], drug carrier [22], bioimaging [27], etc. These days, their composite with polymer is frequently and successfully implemented. The cytocompatibility of Ti_3_AlC_2_, Ti_3_SiC_2_, and Ti_2_AlN was confirmed by Chen et al. [138] theoretically, using DFT calculation. It also made nanocomposites with PLA for bone generation [139]. The n-octyltriethoxysilane (OTES) was used for better adjustment with PLA. As a result, MC_3_T_3_-E1 cells were adhered to get multiplied on the composite membrane [137]. The Ti_3_C_2_Tz (0.75 wt.%)/CS composite nanofibers were synthesized by Mayerberger et al. [137] using electrospinning, and they found the reduction of E. coli and S. aureus by 95% and 62%, respectively, in four hours. The fiber was used as a bandage. Xing et al. [19] found Ti_3_C_2_/cellulose composite hydrogel to be a biocompatible, biodegradable, and load bearer for drugs after their photothermal conversion. They can deliver the anticancer drug doxorubicin hydrochloride (DOX) in at least 48 h. They can scavenge and kill cancer/tumor cells by photothermal and chemotherapy at around 50–55 °C. It represents one of the successful experiments for cancer treatment using MXene/polymer nanocomposites.

**Figure 8:**
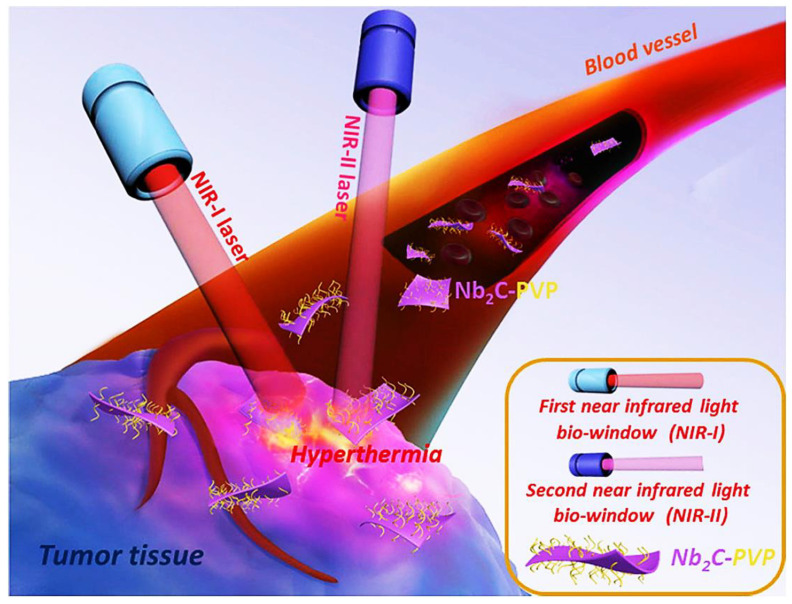
A sketch of Nb_2_C/PVP used for tumor ablation by In Vivo Photothermal irradiated with NIR-I and NIR-II (Reprinted with permission from ref. [140] Copyright 2017 American Chemical Society).

**Figure 9:**
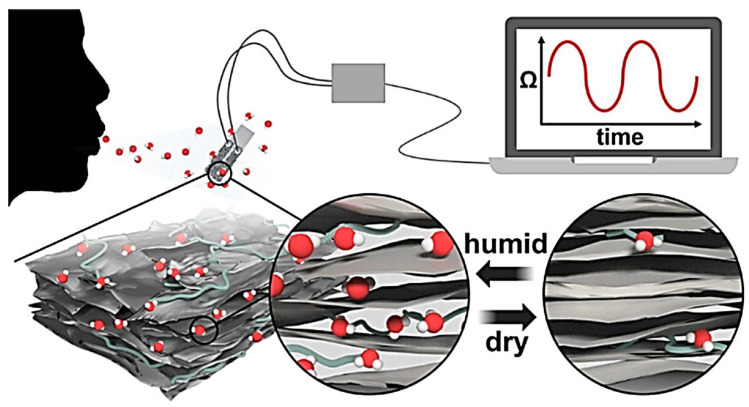
Polyelectrolyte/MXene Multilayers detector of human respiration (Reprinted with permission from Reference [141]. Copyright 2017 American Chemical Society).

Similarly, Lin et al., 2017 [140] found Nb_2_C/polymer nanocomposites to have higher photothermally active material that gives biodegradable 2D material for tumor ablation by a photothermal process in the near-infrared region, as shown in Figure 8. Likewise, MXene/Polyelectrolyte [141] and Ti_3_C_2_T_x_/polyimide nanocomposites were used as humidity sensors to check people’s breathing for the disease diagnosis, as shown in Figure 9. Acetone and ammonia presence indicates the condition of diabetes and lung discomfort, respectively. Human hand and face detection was done easily by an MXene/PVA hydrogel-based flexible sensor developed by Zhang et al. [142]. Through this discovery, the perception sensor for human health was a great achievement in medical science. However, a rigorous and vigorous study is needed for these nanocomposites’ high-level and reliable application.

##### Antimicrobial Agent

The effect on health due to different microbial growth is reduced with different 2D materials [31]. Among them, MXenes are more active than the usually known most active Graphene Oxide. The antibacterial properties of an agent are measured by the permeability of the cell membrane and sharp edges that can rupture the membrane and destroy the bacteria’s DNA [143]. Moreover, the higher conductivity of MXene indicates its more antibacterial properties than Graphene Oxide. For example, Rasool et al., 2017 showed improved hydrophilicity with a contact angle of 37° and reduced the large pores in the membrane [31]. Other antimicrobial action measures are physical stress, roughening of the surface, and cell wall disruption due to the sharp edges of MXene [144]. Mayerberg et al. developed electrospun MXene (Ti_3_C_2_Tz)-chitosan (CS) nanofibers of antibacterial nature used in biodegradable medical bandages. The physical phenomenon is the hydrogen bonding or electrostatic interactions between the negative MXene functional groups and positive nitrogen-containing groups of chitosan [137]. MnOx/Ti_3_C_2_-SP and MnOx/Ta_4_C_3_-SP MXene nanocomposites are used for acidic tumors [20,145]. Ag @ Ti_3_C_2_ @Cu_2_O nanocomposites has photo catalyst appropriate for antibacterial purposes [146]. Thus, polymer coated with MXene act as an antimicrobial agent. 

##### Anticancer Activity

Chemo and radiotherapy are the known anticancer treatments found to be invasive to the body. This limitation was overcome by photothermal therapy (PTT) incorporated with NIR (700–1300 nm). The extinction coefficient is the light absorption performance and is denoted by α; the photothermal stability is light to heat convertibility, denoted by η. The Lambert–Beer law (A/L = αC) gives the extinction coefficient (α). Li et al. showed light absorption by MXene droplet, thereby increasing its temperature. The MXenes, especially Ti_3_C_2_, show a higher absorbing capacity of nearly 100% than the carbon nanotubes [98]. 

Dai et al. in 2017, worked on MnOx/Ta_4_C_3_-SP nanocomposite whose α and η were found to be 8.67 × 10^7^ Lg^−1^nm^−1^ and 34.9% [20]. The near-infrared radiation of wavelength 808 nm and 1064 nm laser induces the hyperthermal radiation at 61 °C and 66 °C and then ablates the tumor cell to kill it. There is no side effect of toxicity from light, indicating their safety and compatibility. The same characteristics were found in MnOx/Ti_3_C_2_-SP [27] with α and η of 5.0 × 10^7^ Lg^−1^nm^−1^ and 22.9% using NIR of 808 nm at 60 °C. The myeloperoxidase (MPO) enzyme is a catalyst that helps in the degradation of toxic cells by transforming the hydrogen peroxide into cytotoxic hypochlorous acid (HOCl^−^) which can kill bacteria and other pathogens. They have a slight adverse effect on the host cell. The metabolism occurs in all healthy tissues in a safe and subtle mode with the addition of (MXene) Nb_2_C/PVP (Polyvinyl Pyrrolidone) composite. The Kupffer cells that are responsible for breaking RBC (with a blood half-life of 3.8 h) break the composite flakes (>200 nm) enclosing the toxic cells into 10–50 nm-sized nanoparticles, which are then excreted from the body. So, Nb_2_C-PVP nanoflakes are biocompatible composites. 

#### 5.1.2. Drug Delivery System

**Figure 10:**
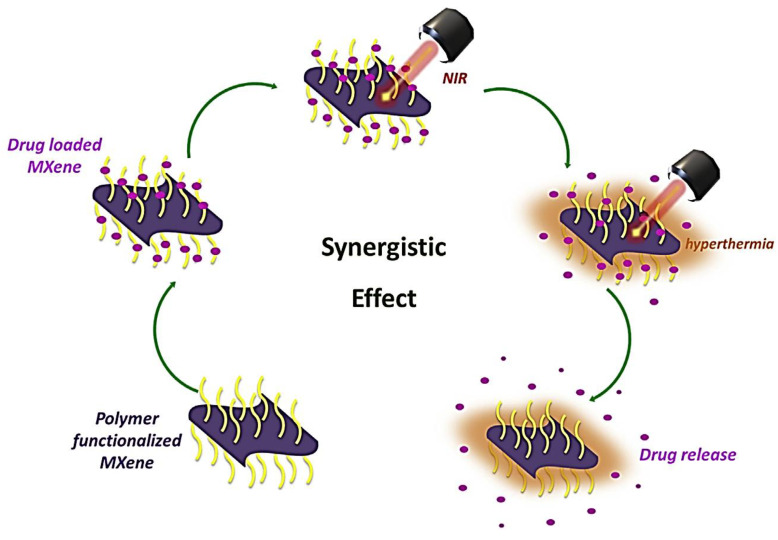
The chemo and photothermal therapy process showing a synergistic effect [11]. Reprinted with permission from Ceramics International. © 2019 Elsevier Ltd. and Techna Group S.r.l.

**Figure 11:**
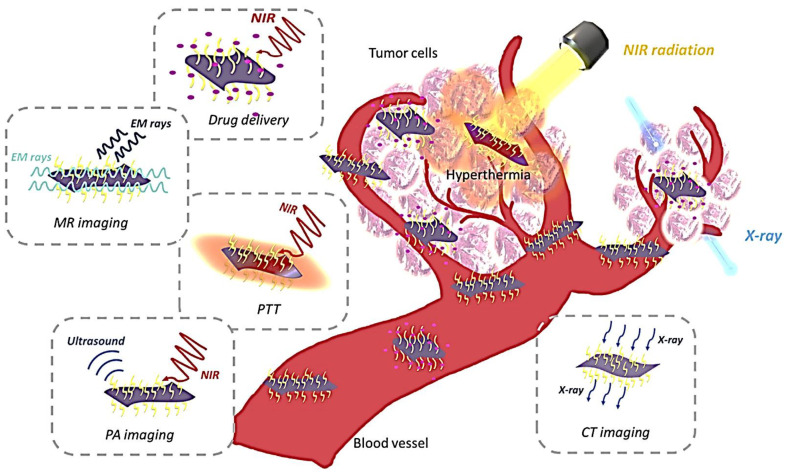
A sketch of PTT/chemotherapy [11]. Reprinted with permission from Ceramics International. © 2019 Elsevier Ltd. and Techna Group S.r.l.

The temperature and certain enzymes affect the tumor cells with low pH values. The conventional drug delivery into the cancer cell is adverse to the non-malignant cells [147]. This can be overcome by making drug molecules positively charged by adding the OH^−^ or F^−^ groups on the surface of the MXene and circulating in the bloodstream [32]. MXenes high photothermal conversion and pH sensitivity result in the synergetic effect of the drug release and malignant cell ablation, as shown in its schematics in Figure 10 and Figure 11 [11], respectively. Han et al. found Ti_3_C_2_ MXene surface-modified SP as one of the examples of such nanocomposite [22] having a 74.6% inhibition rate and ablating temperature of 68.5 °C by NIR laser, thereby destroying the tumor cell by the synergistic effect. The Ti_3_C_2_ MXene-cellulose hydrogel was synthesized by Xing et al. in 2018 and has worked as photothermal and chemotherapy [19]. There was 98% water in the pores of the composite into which 84% of the drug was loaded, which shows the biocompatibility under the irradiation with NIR of 808 nm and reduces toxicity by the drug delivery. It was found that 100% of cancer cells were destroyed in two weeks with the use of the hydrogel MXene of 235.2 ppm irradiated with NIR of the power density of 1.0 W/cm^2^ for 5 min. 

#### 5.1.3. Bio-Imaging

The allocation of the tumor region and PTT monitoring is done by image diagnosis. There are mainly four types of bio-imaging: 

##### Photoacoustic Imaging

Photoacoustic imaging (PIA) is based on the conversion of light into sound, which controls the divergence of photons and lets to be absorbed into the tissues. The tissue that has absorbed light is heated and produces an ultrasonic wave detected by the detector, thereby creating an image [145]. Since MXene has good absorption and photothermal conversion ability, it can act as a good contrast agent. Yin et al. prepared the mixture of MXene, S-nitrosothiol, and mesoporous silica nanoparticles (Nb_2_C-MSNs-SNO), which acts as an excellent photoacoustic agent [148]. 

##### Magnetic Resonance Imaging

Magnetic Resonance Imaging (MRI) is another non-invasive imaging technique with high resolution and contrasting soft tissues without ionizing rays. Liu et al. prepared an efficient contrasting agent using Ta_3_C_2_-MXene and superparamagnetic iron oxide nanoparticles (Ta_3_C_2_-IONP-SP). In addition, it has an excellent photothermal conversion efficiency of 48.6%, sufficient to kill cancer cells [149].

##### X-ray Computed Tomography

To differentiate the wounded and normal tissues in 3D tomography, the nanomaterial of higher atomic number [145] is used as an X-ray computed tomography(CT) contrast agent (XCTCA). Liu et al. investigated the mixing Ta_4_C_3_-MXene and superparamagnetic iron oxide nanoparticles to form Ta_4_C_3_-IONP-SPs nanocomposites for killing the cancerous cells in the breast using this technique. In addition, these tantalum-assisted MXene nanocomposites have a high X-ray attenuation coefficient that can act as a contrast probe to product computed tomography (CT) [17,20,21]. Further, they have a photothermal conversion efficiency of 32.5%, which is sufficient to kill the cells completely [17].

##### Luminescence Imaging

The quantum dots (QDs) derived from MXenes (MQDs), whose size is controlled by the reaction, have excellent photoluminescence characteristics. Xue et al., 2017, synthesized water-soluble Ti_3_C_2_-MQDs. Their PL spectra depend on their excitation energy. It can easily detect Zn^2+^ ions with perfect selectivity [150]. 

#### 5.1.4. Sensors

The MXene-based nanocomposite sensors used in biomedical applications can be of two types:

##### Biosensors

Biosensors are used to detect certain elements in the human body. They are based on optical and electrical signals. The biosensors comprise a sensing element, a transducer, and a data interpreter. The sensing element is an immobilized biomolecule-like enzyme capable of detecting the concentration of the analyte, thereby producing the biochemical signal which is converted into an electrical signal by a transducer and recorded by a data interpreter [151]. Rakhi et al. prepared GOx/Au/Ti_3_C_2_/Nafion/GCE enzymatic biosensor to detect glucose [152]. The glucose is converted into gluconolactone and hydrogen peroxidase (H_2_O_2_) with the help of the enzyme glucose oxidase (GOx). The high potential developed by oxidation/reduction indicates the production of peroxide with the help of the GOx enzyme due to the presence of glucose and hence acts as the glucose sensor. The adhesion of the enzyme with GCE is supported by Nafion [153].

Thus, the polymer MXene nanocomposites are prosperously used in biomedical applications [11] due to their physiological stability, selectivity, biocompatibility, response to stimuli, contrast, NIR absorption, and photothermal conversion sensitivity. MXene is also used for temporary implants, genetics, tissue culture, immunopathology, and fertility treatment. The conducting polymers like PANI and PVP on MXene substrates are used to detect analytes, cancer, and photothermal therapy. These nanocomposites also crush kidney stones. The vigorous study of polymer-based MXene nanocomposite is needed for bio application. 

##### Physical Sensors

Unlike biomolecules in biosensors, the sensors in which pressure and tension change the electrical signals are called physical signals. Thus, these sensors are used for human activity by flexible wearing [154,155,156,157]. Various sensors with higher sensitivity and selectivity are due to better conductivity, lower bandgap, and sites with higher activity [158,159,160]. Electrochemical, photoluminescence, mechanical, and electrochemical types of sensors are now being developed using MXene [149,150,151]. This MXene, when mixed with the polymer, can provide flexible sensors [161] like humidity [162,163,164] and mechanical [165], etc. Materials impedance and water absorption affect the sensitivity of the material. Impedance-like material properties that change with attached, absorbed, or unfiltered water affect the humidity sensors used in the environment, medical care, food, metallurgy, etc. 

**Figure 12:**
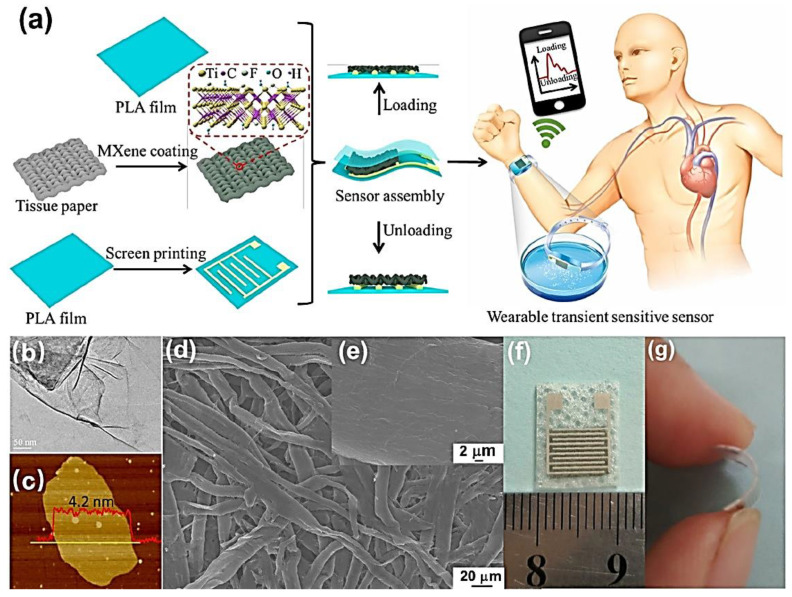
MXene nanosheets-based composites for wearable pressure human sensors (Reprinted with permission from Reference [157] Copyright 2017 American Chemical Society). (**a**) Fabrication of flexible wearable transient pressure sensor using MXene sheets. The (**b**–**e**) represent TEM image of MXene nanosheets, AFM image of MXene nanosheets deposited on mica plate, SEM image of MXene/tissue paper, SEM image of MXene/tissue paper fiber, respectively. The (**f**,**g**) are the photographs of the pressure sensor.

In 2017, a highly sensitive humidity sensor was created by and Kim’s team [162] and Sajid’s team [163] using a 2D Mo_2_C, Cr_3_C_2_, and polymer (PAM, PVA) composite. An et al., 2019 [141] found Ti_3_C_2_T_x_/PDAC humidity sensors to have repeatable sensing properties (Figure 12), and their sensitivity was inversely related to the material conductivity. PDAC stands for poly diallyl dimethyl ammonium chloride and is responsible for improving electrical properties. The sensing efficiency of the composite was increased to 46%, comparable but not more than other commercially found sensors after its deposition time was changed from 5 to 15 min. The change in time increases the interlayer spacing, thereby reducing the conductivity from 47 to 27 S/cm. If the water absorption by PDAC was higher, this limitation would have been overcome. 

Another challenge is detecting the volatile organic compounds (VOCs) that are toxic pollutants found as by-products of industries. Yuan et al. [166] coated PVA/PEI polymer with Ti_3_C_2_T_x_ to produce a VOCs gas sensor with higher sensitivity of 0.10–0.17 ppm. PVA/PEI helped in the adsorption of polar gas molecules by increasing the larger specific surface area. The electrical stability of the sensor was fine even after 5000 cycles after the strain stimulation from 1.2 to 8 mm/s at 80% strain. Zhang et al. [142] investigated the hydrogel strain sensor using MXene/PVA crystal mud composite. A wearable pressure sensor was developed by Guo et al. [157] (as shown in Figure 12) by mixing a degradable PLA and Ti_3_C_2_T_x_ and found superb stability even after 10,000 compression cycles with excellent response and recovery time. 

Hu et al. [167] used chitosan (CS)/Ti_3_C_2_ composite for wearable pressure sensors where CS helped in connecting the interlaminar sheets of MXene. Their response time was fast enough (109.6 ms) with a recovery time of 110.6 ms with higher cycle stability even after 150,000 cycles (Figure 12). Li et al. [168] added polyurethane (PU) in a Ti_3_C_2_@CS to form a pressure sensor. These are examples of how the MXene/polymer nanocomposites are used for sensors of different kinds. Still, more vigorous studies are needed for their optimized and improved application. 

Sensor Parameters and Calculation: Electrical Conductivity, Flexibility, Sensitivity, and Repeatability of Sensors

D. Jang et al. studied wearable sensors with the help of electromechanical sensing capacity with respect to their electrical conductivity, flexibility, sensitivity, and repeatability of carbon nanotubes-carbonyl ironfiller polymer (CNT-CIP) based composites [169] that can record on the basis on vibration and magnetic field intensity. The sensors were tied to human body joints (finger, elbow, shoulder, wrist, ankle, knee, or neck) under a low frequency (5, 10, and 20 Hz). They showed 100% flexibility, repeatability of R^2^ > 0.98, and a gauge factor of 2.2 with the fractional change in resistance of 160% for electromechanical irrespective of the body’s motion of a human body. The repeatability was observed to be excellent even after 500 cycles. The CIP-enabled magnetometer measures the external applied magnetic field strength. The conductive fillers help to maintain the cyclability depending on time as an electrical characteristic. The change in resistance concerning the deformations is recorded through the bridge circuit after amplifying the change with the help of an amplifier [60]. The electromechanical sensing performance depending on stretching and compressing is given by [169],

$$Change\;in\;resistance=\frac{R_{e}-R_{o}}{R_{o}}\times 100\%$$

*R_o_* and *R_e_* are the sensor resistances at initial and stretched conditions.

The fractional change in resistance (FCR) and the applied tensile strain (ε) at the stretched condition are related as, $FCR_{\varepsilon }=0.017\varepsilon^{2}-0.251\varepsilon +6.812$. Similarly, the gauge factor, the change in resistance, and applied tensile strain are related as $GF=\Delta R\times R^{-1}\times \varepsilon^{-1}$.

**Figure 13:**
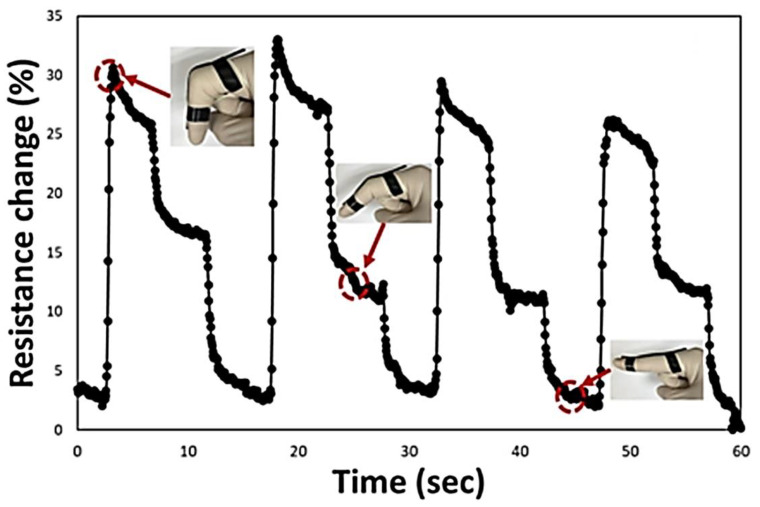
Responses of fabricated sensors during the monitoring of finger gestures in real time for CNT-CIP-based composites [52].

Figure 13 shows fabricated sensors’ responses during real-time monitoring of finger gestures. A similar experiment was carried out on knees and found maximum repeatability and sensitivity needed for observing health care conditions. The responses of other joints are explicitly described by D. Jang et al. [169]. 

**Figure 14:**
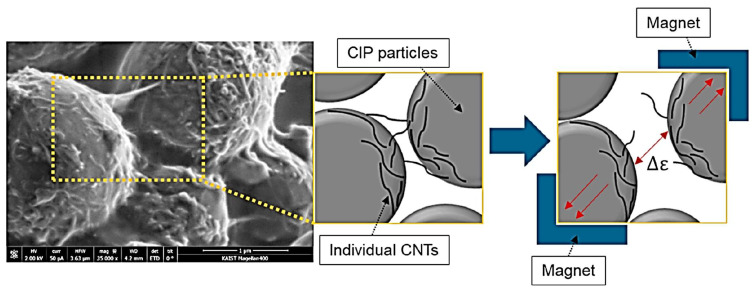
The CNTs and CIP formation schematic under different magnetic fields [52].

They have also checked the sensitivity under different frequencies and magnetic fields. The SEM and other sketchmatic images in Figure 14 show the CNT-wrapped CIP particles. Under the application of a magnetic field (300 mT), there is an increase in the distance of the embedded CNT, thereby changing the conductivity of the network that finally responds to the change due to the magnet field. This way, it can act as a magnetometer device [169].

This CNT@CIP-based nanocomposite was the best among the conducting polymer nanocomposite. However, adding MXene to polymer enhanced the sensing properties exponentially [170]. The incorporation of reduced graphene oxide in the form of spherical polymer particle with MXene by H. Riazi et al. [170] found the sensor with excellent repeatability even after 6000 cycles having a low detection limit of 6Pa and response time of 230 ms. Most of the MXene-based nanocomposites discussed have an efficiency greater than that of CIP@CNT conducting polymers.

Similarly, the drug delivery mechanism performed well with porous organic polymers.

#### 5.1.5. Tissue Engineering 

Like other exotic applications of MXene-based nanocomposites, tissue engineering is an important application. Tissue engineering mainly deals with the engineering of the bone, skin, nerve, and myocardial. Orthodontic implantation and periodontal regeneration is a basic theory for guided bone generation that controls soft tissue interference In Chen et al., a Ti_3_C_2_Tz MXene was mixed with the mixture of PLA matrix and octyltriethoxysilane (OTES) as a mediator. The mechanical properties of the results were increased significantly (33%) along with the increased biocompatibility [171]. This composite help in bone tissue engineering, dressing wound, etc. [172].

Mao et al. prepared electroactive composite hydrogel BC/Ti_3_C_2_ for repairing and regenerating skin. Similarly, Nicolette et al. prepared Ti_3_C_2_ neuroelectronic devices with microelectrode arrays made on a flexible polymeric substrate for appropriate neural tissue engineering. The interface impedance was reduced four times to that of equal-sized gold microelectrodes, the signal-to-noise ratio was higher, and sensitivity was decreased to 60 Hz along with increasing adhering capacity, axons growing, and forming the functional network. Ye et al., 2020, prepared poly (ethylene glycol) diacrylate (PEGDA)-gel-methacrylic anhydride (MAA) cryogel for the treatment of myocardial infarction (MI). 

#### 5.1.6. Therapeutics

The surgical method of cancer causes larger wounds which further causes infection. The chemotherapy method kills the cancerous cell and the other normal cell. For treatment of only the cancerous cell, different therapy methods are developed. 

##### Photothermal Therapy

In this method, the tumor cell is ablated with the absorbance of near-infrared rays, which develop heat on the site. The basic properties of MXene-like hydrophilicity, larger surface area, higher photothermal conversion, etc., make it an appropriate material for such purposes. In vivo PPT depends on the depth of the tumor tissue and laser penetration along with the PPT agent absorption, time of accumulation, and power density of light. Lin et al. prepared an organic-inorganic composite of PGLa/Ti_3_C_2_ and found higher efficiency for photothermal conversion [173]. Ti_3_C_2_/Al treats cancer under 808 nm laser radiation [17]. V_2_C nanosheets are effective photothermal agents (PA) for photothermal treatment with PA and MRI [174]. 

##### Photodynamic Therapy

Photodynamic therapy (PDT) is another method for cancer treatment without side effects. The effective electrical and optical properties of MXene can activate reduced oxygen species (ROS), which can kill the cancer cell. The PDT combined with PTT can effectively be used in cancer treatment. Ti_3_C_2_-DOX composite can generate ROS in photodynamic therapy that kills cancerous cells [175]. They are also used in drug delivery. Nb_2_C/polymer nanocomposites ablate tumors by photothermal processes around near-infrared regions [140].

##### Thermodynamic Therapy

Since tumorous cells are deficient in oxygen in most cases, it might be difficult to activate ROS at the site. In that condition, Xiang et al. forwarded the concept of free radicals produced by the thermally unstable initiators like Azo Initiators with Propane and Hydrate (AIPH), which generate heat to kill the cancer cell without an oxygen environment. They prepared PLGA/Ti_3_C_2_ AIPH@Nb_2_C@mSiO_2_ nanocomposites used for thermodynamic therapy to kill cancer cells deficient in oxygen [176]. 

## 6. Energy Applications

The numerous redox sites, brilliant conductivity, and larger surface area make MXene an ideal material for energy storage [177,178,179,180]. It is better than any other carbon electrode due to its capacitive density higher than 900 F cm^3^ [181]. However, the piling of MXene destroys their exotic properties like ion diffusion, intercalation, adsorption, and sites for the reaction. These problems are overcome by the addition of polymer to form nanocomposites and used to prepare highly efficient capacitors called supercapacitors which are abundantly used in photo and electrothermal applications [182,183]. Most of the electroactive polymers [177,179,180], such as polyfluorene derivatives (PFDs), poly(3,4-ethylene dioxythiophene) (PEDOT), and its derivatives [184,185,186], PPy [103], PANI [187], PVA [9], and PDAC [9] are used to form the nanocomposites used in energy storage applications.

Gogotsi’s team has wider research in this field. They [9] investigated MXene/PVA-KOH nanocomposites. Due to the extended interlayer spacing, their volumetric capacitance of 80% is much higher than that of the MXene/PDAC composite. The PVA assisted in the improvement of ion mobility charge transportation. Boota et al. [188] found MXene/PFDs nanocomposites to have 1.5 times higher capacitance density than the intrinsic one and nearly equal to MXene/PPy [181].

Qin et al. [184] used PEDOT: PSS as an intercalator of Mo_1.33_C MXene to prepare a supercapacitor with the capacitance of 568 F cm^−3^. The addition of concentrated H_2_SO_4_ increases the capacitance to the highest value of 1310 F cm^3^ reported to date. The increase in the conductivity of polymer has helped in increasing the capacitance. Simultaneously, the recycling capacitance of 92% of the initial is found even after 25,000 cycles. Increasing conductivity decreased the electron transport interlayer resistance. This mechanism creates the way for the movement of ions. The oxidation on the surface of the MXene also positively contributes to capacitance [181]. In addition, Qin et al. [189] studied nanopores’ effect on the electrode in the resultant composite. 

Wu et al. [102] and Zhu et al. [103] studied the double layer and pseudo capacitance of MXenes and PP. The capacitance and wettability increased in the resultant composite [187]. The nanocomposites electrode of MXene and polymer (Ti_3_C_2_/PEDOT: PSS) for high frequency was studied by Gund et al. [186] (Figure 14). They found that the high frequency (120 Hz) capacitance of 24.2 F cm^3^ with working ability and life of 60–10,000 Hz and its working life was greater than 50,000 h, nearly equal to the commercial capacitor. The MXene polymer nanocomposites are used in the supercapacitor but are much rare in batteries [123]. 

Chen et al. [119] prepared MXene/PEDOT by self-initiated polymerization and was found to have improved lithium storage capacity, and efficiency increased from 38% to 53%. Dong et al. [190] synthesized Ti_3_C_2_/PP and prepared its cathode for the LiS battery without a metal collector [191,192]. Their capacitance was about 1062 mAh/g at a 0.2 C charge rate in a reversible mode. The solid electrolytes for solid-state electrochemical cells were prepared from these composites. Fei et al. [193] mixed MXene with PBI to separate the fuel, which enhances the strength, conductivity, and resistance of the PBI membrane. The MXene/polymer nanocomposites are used in energy storage and energy conversion like photothermal and electrothermal conversion used in wearable heaters, anti-froster, self-healing, etc. The MXene/polyethylene glycol (PEG) was developed by Fan et al. having 94.5% storage efficiency [194,195,196,197]. It is more or less green synthesis of energy next to alternative methods like solar cells [198,199,200].

With the development of miniaturized electronic devices used in every field of society, the development of multifunctional supermicrocapacitors is essential [201]. The use of MXene polymer-based nanocomposites in such capacitors is described as follows.

### 6.1. MXene-Polymer-Based Micro-Supercapacitors

Unlike the conventional method, the 2D Mo_1.33_C MXene can be mixed with ethylene dioxythiophene (EDOT) in the presence of the electrochemical polymerization of organic monomers whose electrochemical behavior was similar to EDOT/Na_2_SO_4_ electrolyte solution (E-N). The electrode film is increased with the peak current out to the EDOT radical cations [202,203,204]. The oxidizing potential of EDOT-Mo_1.33_C MXene (E-M) is E_onset_ = 0.76 V vs. Ag/AgCl lower than E_onset_ = 0.89 V vs. Ag/AgCl of E-N. This difference is due to the anomalous surface charges and edge defects of MXenes. Similarly, Mo_1.33_C MXene with Pyrrole (P-M) and Ti_3_C_2_ MXene with EDOT (E-T) or pyrrole (P-T) were studied and were found to be stable concerning their zeta potential. Here, the electrolyte is not used for electroplating. Instead, the electrolyte is the negatively charged MXene (Ti_3_C_2_, Mo_1.33_C), for which it gets electrons from the organic monomers, couples each other, and forms polymer chains. The MXene then moves towards the electric with the help of an electric field, adsorbed into the polymer chain, and results in the complex film as a highly conductive electrochemical performer. The images of four films (E-N, E-M, P-S, P-M) were described and verified with the help of SEM, XPS, etc., as described by L. Qin et al. [189]. 

**Figure 15:**
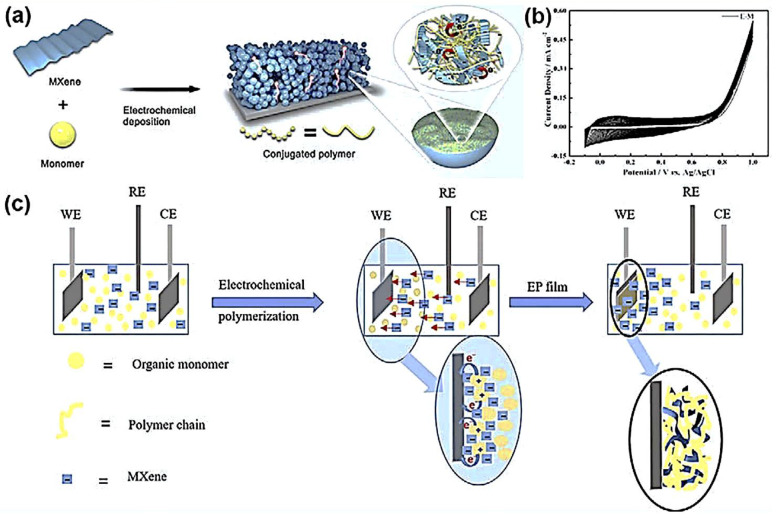
(**a**) The electrochemical polymerization of conjugated polymer-MXene composite. (**b**) Cyclic voltammogram of (**a**) V with the scan rate of 50 mV s^−1^. (**c**) MXene-facilitated electrochemical polymerization for flexible solid-state microsupercapacitors. Reprinted with permission from Reference [189] @ 2019 Elsevier Ltd.

The electrodes can improve the stagnant ion transport in electrolytes through the electrodes in pseudo-capacitors with pores [37,38,39]. Further, the conductivity and the capacitance of the MXene polymer composite were increased by the dopant used. The in-plane solid-state micro-supercapacitors (MSCs) are prepared by in-situ EP on the patterned electrode in steps, as shown in Figure 15a. 

In the electrochemical polymerization process, the environment of electrolyte is provided by the negatively charged MXene (Ti_3_C_2_, Mo_1.33_C) and organic monomers (Pyrrole, EDOT). A polymer chain is formed between organic monomers and electrodes after losing loose electron electrons to the electrode. On the other hand, the MXene also moves towards the electric field-driven working electrode (WE) and forms a complex film, as shown in Figure 15c. The counter electrode (CE) and reference electrode (RE) are kept for controlling the electrochemical current and proving standard value to the system. The rectangular cyclic voltammogram curves [189] obtained show their low resistance and good reversibility (Figure 15b). The peak current is increased in every cycle, indicating the coupling of the EDOT cations and film growth on the electrode. The oxidation takes place with a smaller voltage (0.76 V) in EM than that of EN (0.89 V). N stands for Na_2_SO_4_. The areal capacitance of the electrode material from MXenes solution is higher than that from conventional electrolytes due to the porous structure of the MXene. The electron enters inside the film with the highly conducting MXene peripheral shell. The electrochemical performance of the four microsupercapacitors E-M shows a smaller charge-transport semicircle than E-N MSC. The composite film EM and PM conductivities are higher than polymer film EN and PS [189]. 

### 6.2. Asymmetric Microsupercapacitors (AMSC)

The use of MnO_2_ (cheap, eco-friendly, with a high specific capacitance of 1375 Fg^−1^ transitional metal oxide) [204,205,206] as a positive electrode and EDOT-Mo_1.33_C MXene (E-M) as the negative electrode gives rise to an asymmetric microsupercapacitor. The control of deposition time on positive and thickness on EM film negative give rise to the widest voltage window of about 1.6 V MXene-based microsupercapacitors [207]. The pseudo rectangular CV consistent curve, even at a high scanning rate of 1000 mVs^−1^, shows their low resistance and fast reversibility as the condition of supercapacitors similar to E-M MSCs. After 10,000 cycles, their internal resistance is slight, and the microscopic structure is hardly changed. The galvanostatic charge/discharge cycles show higher stability with 92% capacity after 10,000 cycles. The energy and power density of E-M MSCs is found in the range 18.7–20 mWh cm^−3^ and 0.4–8.3 W cm^−3^, respectively, and that for P-M are 5.9 to 11.6 mWh cm^−3^ and 0.3–6.8 W cm^−3^, respectively. The MSCs based on other materials like carbon materials have an energy density of 0.15–9 mWh cm^−3^ which is less than that of EM MSCs and PM [208,209,210,211,212]. Likewise, the transition metal oxides/hydroxides/carbides have 1–5 mWh cm^−3^ [213,214,215,216,217,218] and conducting polymers have 5–11 mWh cm^−3^ [219,220]. The areal capacitance and energy density achieved were 69.5 mF cm^−2^ and 250.1 mWh cm^−3,^ respectively. 

**Figure 16:**
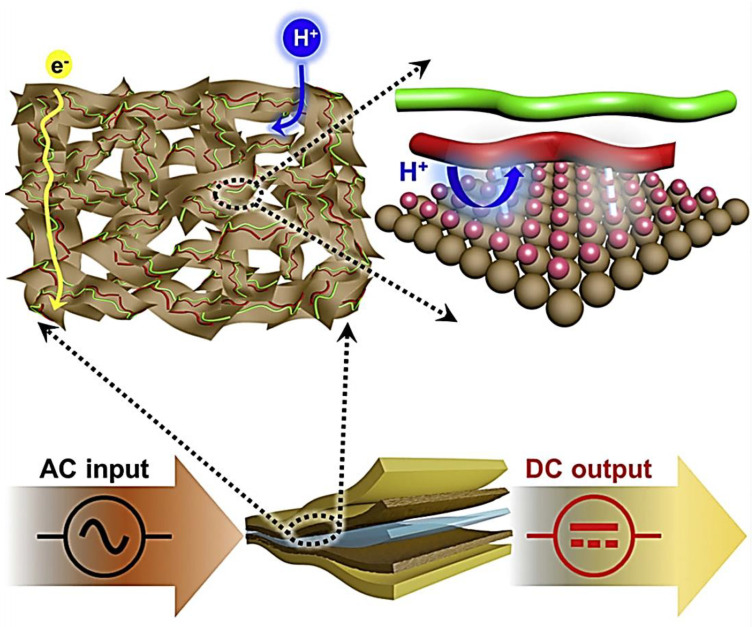
Energy storage devices (asymmetric supercapacitors) based on MXene/(PEDOT: PSS) nanocomposites for high-frequency application (Reprinted with permission from Reference [186] Copyright 2019 Elsevier B.V.).

This way, very convenient and concise methods synthesize conjugated polymer MXene composites following in situ EP without using electrolytes. The applied current on the electrode can control the film’s polymerization and thickness, which are applicable for the synthesis of ASMCs. These microcapacitors are used in portable electronic devices, electromechanical, and robotics on the nanoscale. The porous MXene/(PEDOT: PSS) used in energy storage devices for high-frequency applications is shown in Figure 16 [186]. The gray sheets are the Ti_3_C_2_; the red fibers are of PEDOT, and the green fibers are of PSS, over which the transport of electrons (e^−^) takes place. The interaction among the composite oxidizes benzoid to quinoid. The filtering of AC was at a flexible and large scale with volumetric capacitance @1000 Vs^−1^ at a frequency of 10,000 Hz over 30,000 cycles.

## 7. Conducting Polymers Nanocomposites vs. MXene-Polymer Nanocomposites

Sharma et al., 2021 mentioned several drawbacks of these conducting polymer nanocomposites. They have toxic-carcinogenicity that leaves disparities in vivo and vitro studies. They have many limitations in biocompatibility, bioactivity, cytocompatibility, and other physical characteristics. Further, they have hydrophobicity and have an initial overflow drug release rate which greatly affects the drug delivery mechanism for several biomedical treatments like cardiovascular, neuro, cancer, etc. Similarly, they have lower cyclic stability, specific-capacitance, power-density, and energy capacity than carbon and other oxides of transitional metals [221]. 

These drawbacks are overcome by using MXene with those polymers superficially or compositionally in the nanocomposite form. According to Riaz et al., 2014, the conductivity of the conducting polymer composite, especially polyaniline (PANI), is in the order of 10^−5^ to 10^−4^ S/cm. The metallic conductivity of MXenes viz Ti_3_C_2_T_z_ is found to be in the order of 10,000 S/cm [160]. They can improve electromagnetic and radio frequency shielding [222], create strain, gas, selective molecule sensors, and energy conversion and storage mechanisms. Naguib et al., 2016 found the penetrating network for conducting path needed for delivery systems termed as percolation threshold to be 1.7 vol% (volume fraction of MXene PAM/Ti_3_C_2_) [126]. They show their resistance depending on temperature. On the other hand, the percolation threshold of CIP@CNT-embedded polymeric composites ranged from 0.5% to 0.75% [169].

The intrinsic conducting polymers have complicated microstructures that affect the reproducing and controlling of doping level stability of the nanotubes/wires. The fabrication or assembling into integrated chips is quite complicated, unlike MXene polymer nanocomposites. Thus, incorporating MXene into the intrinsic or extrinsic polymer composites will likely overcome the conducting polymer’s drawbacks alone.

An updated comparative list of different MXene-polymer nanocomposites is presented in Table 1 and the MXene-based nanocomposites vs. other polymer nanocomposites of the same field are presented in Table 2 respectively.

## 8. Summary and Outlooks

In the way of development of 2D material, MXene is discovered from its parent MAX phase after etching out the A element. MXene is found to have a larger areal surface, metallic conductivity, and hydrophilicity with tunable size. Previous studies have shown that after mixing MXene with polymer, the resulting nanocomposite possesses various excellent functional properties. The surface processing of polymer with MXene makes a polymer stable physiologically using NIR absorption through photothermal conversion. The high response to the stimuli and enhanced sensible resolving power to biological processes lead the MXene-based polymer composites towards several biomedical applications. Due to the excellent electrochemical properties of MXene, it can be considered an alternative to the previous electrode material for batteries and supercapacitors.

Despite the advantages and multifold applications, the large-scale production and industrial applications of MXene/polymer composite have to overcome several challenges. For example, the chemical etching route used to produce MXene requires toxic chemicals, which hinder the ecofriendly production of MXene. The dispersion of MXene in a hydrophobic polymer is also a challenge in MXene/polymer composite preparation. It can be believed that as the field of MXene/polymer composites is developed further, there will be much progress on the scalable and ecofriendly production of MXene. Moreover, its effective exfoliation and homogenous dispersion in various polymers should be realized for various potential applications. The synthesis through the formation of clay is another low-cost approach. After reforms on the mentioned limitations, the cost of MXene production will be lower than that for exotic nanofillers like carbon nanotubes.

## Figures and Tables

**Table 1:** Summary of MXene-Polymer Nanocomposites. Adopted with the permission from Ref. [223], European Polymer Journal © 2019 Elsevier Ltd. All rights reserved.

SN	MXene	Polymer	Result	Application	Ref.
1.	Ti_3_C_2_T_x_	PVB	RLmax value of −46.3 dB at 5.8 GHz	EMIS	[224]
2.	Ti_3_C_2_T_x_	UHMWPE	Addition of Ti_3_C_2_ increases antifriction properties, mechanical strengths, and crystalline property	Improving mechanical properties	[225]
3.	Ti_3_C_2_T_x_	PES	Gentian Dye with flux 117.6 9 Lm^−2^h^−1^ rejects 80.3% and that with 114.9 Lm^−2^h^−1^ rejects 10.7% at pressure of 0.1 MPa.	Ultrafiltration membranes for purification	[83]
4.	Ti_3_C_2_T_x_	Chitosan	Recover 94–105% for malathion recovery in tap water.	Biosensor	[226]
5.	Ti_3_C_2_T_x_	Cellulose Nano fibers	EMIS ~25.8 dB at 12.4 GHz with 80% of d-Ti_3_C_2_T_x_ and ρ ~739.4 S m^−^^1^.	EMIS	[93]
6.	Ti_3_C_2_T_x_	PS	Improved electrochemical performance.	Immobilization of soluble PS	[95]
7.	Ti_3_C_2_T_x_	PS	Capacity reduced 0.05%/cycle, the SC of 1200 mAhg^−1^ over 5 h. C/DC current rate and a CRR of 80% attained over 400 cycles at 2 h. C/DC current rate.	Supercapacitor	[96]
8.	Ti_3_C_2_T_x_	PVDF	The antibacterial rate of the fresh membrane reached 67% and 73% compared to that of PVDF, while aged membranes exhibited over 99% growth inhibition.	Anti-fouling ultrafiltration membrane	[31]
9.	Ti_3_C_2_T_x_	PVDF/PDMS	Highly efficient light-to-heat conversion rates at nearly 100%.	Photothermal conversion	[98]
10.	Ti_3_C_2_T_x_	P(VDF-TFE-CFE)	~15 wt.% MXene raised dielectric permittivity to 105 and 10 wt.%. MXene raised the dielectric constant 25 times.	Enhanced electric properties	[99]
11.	Ti_3_C_2_T_x_	Polypyrrole	Attained maximum SC of 184.36 Fg^−1^ at 2 mVs^−1^ with CRR of 83.33% after 4000 charging cycles at 1 Ag^−1^	Supercapacitors	[102]
12.	Ti_3_C_2_T_x_	PVA/PAA	Composite nanofibers displayed excellent catalytic activity against 4-NP.	Wastewater treatment	[104]
13.	V_2_C	PDMAEMA	Increasing temperature from 25 °C to 45 °C increases the transmittance from 15% to 75%, and further addition of CO_2_ increases conductivity from 2.8 to 33.7 mS cm^−1^.	Responsive polymers	[107]
14.	Ti_3_C_2_T_x_	Polyurethane	0.5 wt.% of MXene addition increases the stress by ~70%, tensile strength by ~20%, Pus hardness by ~10%, breaking elongation reduction by ~17%, and water absorption reduction by 10%.	Mechanical properties improvement	[111]
15.	Ti_3_C_2_T_x_	Polyaniline	1:3 mass ratio shows microwave absorption of −56.3 dB at 13.80 GHz with an efficiency of 99.9999%.	Microwave absorption	[114]
16.	Ti_3_C_2_T_x_	P (3,4 EDOT: PSS)	The addition of 1M H_2_SO_4_ gives an excellent result of 1065 F cm^−3^ volumetric capacitance at 2 mV s^−1^.	Increase in volumetric capacitance for ASC.	[115]
17.	Ti_3_C_2_T_x_	Low density polyethylene	Better thermal stability of composites after the incorporation of MXene.	Study of thermal stability	[118]
18.	Ti_3_C_2_T_x_	P-3,4 EDOT	The C/DCC in the first cycle is 575 and 307 mA h g^−1^. After 100 cycles of charging and discharging, the capacitance was maintained at 83% with respect to its first cycle	Upgrade in Li-ion battery technology	[119]
19.	Ti_3_C_2_T_x_	Polyester	Made yarn with SC of 18.39 m F cm^−2^ at 5 mV s^−1^, a power density of 0.39 mW cm^−2^, and a power density of 0.38 μW h cm^−2^. The retention performance was 98.2% over 6000 cycles.	Gave yarn for wearable electronics devices.	[122]
20.	Ti_3_C_2_T_x_	P(3,4-EDOT): PSS	70 wt.% MXene made the fiber with 1489 S cm^−1^ conductivity, 7.13 Wh cm-3 energy density, and 8249 mW cm^−3^ power density.	Conductive fibers	[116]
21.	Ti_3_C_2_T_x_	Polyacrylamide	The conductivity is increased to 3.3 × 10^−2^ S m^−1^ after the addition of 6 wt.% MXene onto the membrane.	Improved flexibility and conductivity	[126]
22.	Ti_3_C_2_T_x_	PEA/P(DMS)	PDMS and PEI membranes are good for non-polar and polar solvent systems. Large-sized PEG addition will enhance their rejection ability.	Solvent resistant nanofiltration in alcohol-based mixtures	[123]
23.	Ti_3_C_2_T_x_	GdW10-based Polyoxometalates	Eradicated tumor cell with Ti_3_C_2_ NSs as a contrast agent for contrast-enhanced CT and MR imaging.	CT/MRI-guided precise PTT of tumors	[24]

**Table 2:** MXene-based nanocomposites vs. other polymer nanocomposites of the same field Adopted with the permission from Ref. [223], European Polymer Journal © 2019 Elsevier Ltd. All rights reserved.

F *	MXene	Other Polymer Nanocomposites
**Biomedicine**	Ta_4_C_3_-IONP-SP nanocomposites are one of the examples used for MRI [17]. Ti_3_C_2_-SP, Ta_4_C_3_-SP, and MnOx/Ti_3_C_2_-SP are used for a photoacoustic signal method with the help of stress waves received from the irradiated tissues by NIR. Ta_4_C_3_-IONP-SPs and Ta_4_C_3_-SP nanocomposites can attenuate X-rays and are used in computed tomography (CT) [17,20,21]. Ti_3_C_2_ +colloidal solution allows the growth of Gram (−) *E. coli* and Gram (+) Bacillus subtilis [67] antibacterial growth. Ti_3_C_2_Tz + PLA + octyltriethoxysilane (OTES) has good mechanical properties and biocompatibility help in tissue engineering [171,172]. (GOx/Au/Ti_3_C_2_/Nafion/GCE) an enzymatic biosensor that detects glucose [152]. Therapeutics: PLGA)/Ti_3_C_2_ [173] used in cancer treatment by photothermal ablation. Ti_3_C_2_/Al treats cancer under 808nm laser radiation [17]. V_2_C nanosheets are effective photothermal agents for photothermal treatment with PA and MRI [174]. AIPH@Nb2C@mSiO_2_ nanocomposites were used for thermodynamic therapy to kill cancer cells deficient in oxygen [176]. (Ti_3_C_2_-DOX) generate ROS in photodynamic therapy that kills cancerous cells [175]. They are also used in drug delivery. Nb_2_C/polymer nanocomposites ablate tumors by photothermal processes around the near-infrared region [140]. MnOx/Ti_3_C_2_-SP and MnOx/Ta_4_C_3_-SP MXene nanocomposites are used for acidic tumors [20,145]. Ag @ Ti_3_C_2_ @Cu_2_O nanocomposites has photo catalyst appropriate for antibacterial purposes [146].	PPy/poly(D, L-lactic acid) with conductivity 5.65 × 10^−^^3^ to 15.56 × 10^−^^3^ S/cm is nerve tissue regeneration (in vivo rat), biocompatibility (PC12 cells) and is used for synthetic nerve conduits [227]. PPy/hyaluronic acid with a conductivity of 3.08 × 10^−^^3^ Scm^−1^ can support tissue growth, stimulate specific cell functions, and be used for tissue engineering and wound-healing applications [228]. PPy nanoparticles/PU with maximum conductivity of 2.3 × 10^−^^6^ Scm^−1^. Cytocompatible with C2C12 myoblast cells, elastomeric properties tissue engineering [229]. PAni nanofibers/collagen with a conductivity of 0.27 Scm^−1^ is well suited for culture and is used as Scaffold material for biomedical applications [230]. PPy/chitosan (10^−^^3^–10^−^^7^ Scm^−1^) have radical scavenger property and is used for food packaging and biomedical applications [231]. PEDOT/glycol (1486 Scm^−1^) and PPy/cellulose acetate (6.9 × 10^−^^4^ to 3.6 × 10^1^ Scm^−1^) used as implantable devices [232,233]. Poly (acrylic acid)/polyvinyl alcohol (0.04–0.06 S/cm) with hydrogel, biocompatible, good mechanical strength, and good swelling properties [234]. Polythiophene derivative/PU with a conductivity of 2.23 × 10^−^^5^ (S/cm) is suitable for supporting electrically stimulated cell growth tissue engineering [235]. Hollow polymeric nanosphere-supported imidazolium-based ionic liquids (HPL-ILs) show enhanced antimicrobial activities [236]. Poly (-L-glutamic acid (PGA) based nanomaterials are highly efficient for drug delivery [237]. The porous organic polymers (POPs) are highly efficient for drug delivery [238].
* Physical/Biosensors	AChE/CS/Ti_3_C_2_T_x_ biosensors detect organophosphates in water and food. AChE/CS-Ti_3_C_2_T_x_/GCE biosensors indicated 94–105% malathion recovery [83] and detect the glucose level in diabetic patients, pollution monitoring, food processing, etc. CS/Ti_3_C_2_ [167] shows a fast response time (109.6 ms) with a recovery time of 110.6 ms and higher cycle stability even after 150,000 cycles. Similarly, Mo_2_C, Cr_3_C_2_, and polymer (PAM, PVA) composites [163], Ti_3_C_2_T_x_/PDAC [141] were good humidity sensors. MXene/Polyelectrolyte [141] and Ti_3_C_2_T_x_/polyimide nanocomposites were used as humidity sensors for breath checking for diagnosis. MXene/PVA hydrogel flexible sensor can detect diabetes indicated by the presence of Acetone and ammonia [142].	CNT@CIP-based nanocomposites show a good sensing property at a low frequency (5, 10, and 20 Hz), showing 100% flexibility, repeatability of R^2^ > 0.98, and gauge factor 2.2 with the fractional change in resistance of 160% and excellent repeatability even after 500 cycles [169]. Pani/BC hydrogel-type composites have a conductivity of 10^−^^2^ (S/cm) and are applicable for biosensors and tissue engineering [88]. PEDOT: PSS/PU (aqueous dispersion) ~120 High pressure sensitivity electronic skin sensor [93]. PEDOT: PSS/PU/ionic liquid 8.8 × 10^−^^5^ (S/cm) are mechanically flexible, stretchable and actuating devices [91].
**Supercapacitors**	Capacity reduced 0.05%/cycle, the SC of 1200 mAhg^−1^ over 5 h. C/DC current rate and a CRR of 80% reached over 400 cycles at 2 h. C/DC current rate [96]. Attained maximum SC of 184.36 Fg^−1^ at 2 mVs^−1^ with CRR of 83.33% after 4000 charging cycles at 1 Ag^−1^ [102]. Adding 1M H_2_SO_4_ gives an excellent result of 1065 F cm^−3^ volumetric capacitance at 2 mV s^−1^ [115].	rGO/Zn/PCz nanocomposites have an improved capacitance of 33.88 F/g [239]. N-doped graphene/Fe_2_O_3_ nanocomposites exhibited a 354 F/g at a current density of 20 A/g [240]. PANI/SWCNT has a capacitance of 485 F/g [241]. La_x_Sr_1−x_Cu_0.1_Mn_0.9_O_3−δ_ (0.3 ≤ x ≤ 1) at 2 A/g current density displayed a capacitance of 464 F/g and that at 64.5 Wh/kg 2 A/g displayed a power density of 2 kW/kg [242].
**EMIS**	PVB/Co2Z/Ti_3_C_2_ has RLmax of −46.3 dB at 5.8 GHz and below −10 dB at 1.6 GHz [224]. EMIS 25.8 dB at 12.4 GHz with 80% of d-Ti_3_C_2_T_x_ having ~739.4 S m^−1^ conductivity [93].	The conductivity of BC/GE/PANI is 1.7 ± 0.1 S/cm [243]. MWCNT/SBR exhibited a shielding efficiency of 35.06 dB [244]. PP/PC/MWCNT shows shielding of 54.78 dB at 0.33 S/cm conductivity [245].
**Conducing Fibers**	Made yarn with SC of 18.39 m Fcm^−2^ at 5 mV s^−^^1^, 0.39 mW cm^−2^ power density with 0.38 μW h cm^−2^ energy density. The retention performance was 98.2% over 6000 cycles [122]. 70 wt.% MXene made the fiber have ~1489 Scm^−1^ conductivity, 7.13 Wh cm^−3^ energy density, and 8249 mW cm^−3^ power density [116].	Carbon hollow fibers show a 287 F/g capacitance at 50 mA/g with CRR 86.4% at 1 A/g [246]. LaNiO_3_ constituted nanofibers ~160 F/g capacitance at ~10 mV/s [247]. GE/PANI nanofiber 976 F/g capacitance at 0.4 A/g current density [248].
**Energy Storage**	The C/DCC in the first cycle is 575 with 307 mA h g^−1^, of which 83% is maintained after 100 cycles [119]. V2O5/MXene shows SC of 768 F/g (at 1 A/g), a specific capacity of 93.3% after the 6000 GDC test, and increased current density from 1 to 5 A/g [59]	SiOx/Fe_3_O_4_/FLG has a CRR of 81.8% valued at 833.4 mAh/g (1550 mAh/cm^3^) at a current density of 0.5 A/g after 500 cycles [249]. SnO_2_/rGO nanocomposites show a CRR of 318 mAh/g at a current density of 500 mA/g after 300 cycles [250].

## Data Availability

Not applicable.

## Abbreviations

CVD: Chemical vapor deposition; PE: Polyethylene; PVA: Polyvinyl alcohol; PU: Polyurethane; EP: Epoxy; PCL: Polycaprolactone; PEO: Polyethylene oxide; PFS: Polyfluorenes; PBI: Polybenzimidazole; PAM: Polyacrylamide; PVDF: Polyvinylidene fluoride; PEI: Polyethyleneimine; PS: Polystyrene; PAAc: Polyacrylate acrylic resin; PLA: Polylactic acid; DMF: N, N-dimethylformamide; DMSO: Dimethylsulfoxide; DMAc: Dimethylacetamide; CTAB: Cetyltrimethylammonium bromide; PANI: Polyaniline; PPy: Polypyrrole; PT: Polythiophene; PDA: olydopamine; PA: Polyamide; LBL: Layer-by-layer self-assembly; MWCNT: Multi-walled carbon nanotube; PI: Polyimide; SA: Sodium alginate; CS: Chitosan; PDMAEMA: Poly(2-(dimethylamino)ethyl methacrylate); PMMA: Polymethyl methacrylate; LLDPE: Linear low-density polyethylene; CNC: cellulose nanocrystals; CNF: cellulose nanofiber; PEDOT: Poly (3,4-ethylenedioxythiophene); PSS: Polystyreneslufonate; TPU: Thermoplastic polyurethane; UHMWPE: Ultra-high molecular weight polyethylene; PAN: Polyaniline; OTES: n-Octyltriethoxysilane; FLIT: Final light-induced temperature; PDAC: Polydiallyldimethylammonium chloride; PP: Polypropylene; LITFSI: Bistrifluoromethanesulfonimide lithium salt; PNIPAM: Poly(N-isopropylacrylamide); PET: Polyethylene terephthalate; PEG: Polyethylene glycol; TBPC:Tetrabutylphosphonium chloride; PDMS: Polydimethylsiloxane; SRNF: Solvent resistant nanofiltration; PFDs: Polyfluorene derivatives; EMI: Electromagnetic interference; LDH: Layered double hydroxide; PVB: Polyvinyl butyral; PAA: Poly(acrylic acid); DOX: Doxorubicin hydrochloride; VOCs: Volatile organic compounds; AgNWs: Silver nanowires; AgNP: Plasmonic silver nanoparticle; PM_2.5_: the mass per cubic meter of air of particles with a size (diameter) generally less than 2.5 micrometers (mm); SEM: Scanning electron microscopy; MD: Molecular dynamics; Eg: Bandgap; DFT: Density functional theory; UV: Ultraviolet light; NIR: Near infrared light; Tg: Glass transition temperature; 2D: Two dimensional GCE—Glassy Carbon Electrode, ROS—Reactive Oxygen Species, AIPH—Azo Initiators with Propane and Hydrate, EMIS—EMI shielding, PVB—Polyvinyl Butyral, PES—Polyether Sulfone, PS—Polysulfide, C/DC—charge/discharge, CRR—Capacity Retention Rate, SC: Specific Capacitance, PVF: Poly (vinylidene fluoride), PDMS—Poly (dimethylsiloxane), P(VDF-TFE-CFE)-Poly (vinylidene fluoride-trifluoro-ethylene-chlorofluoroethylene), PDMAEMA—Poly [2-(dimethylamino)ethyl methacrylate] P(3,4 EDOT: PSS)-Poly (3,4-ethylenedioxythiophene-Polystyrene sulfonate), PEA/P (DMS): Polyethyleneimine/Poly (dimethylsiloxane), C/DCC: Charging/Discharging Capacitance, MWCNT—Multi-walled Carbon Nanotube, BC/GE/PANI—Bacterial cellulose/graphene/polyaniline, SBR—Styrene-butadiene rubber, PC—Polycarbonate.
